# Dual Core-Shell Loaded Lipid-Polymer Hybrid Nanoparticles as Combination Anti-Infective Delivery Platforms

**DOI:** 10.3390/pharmaceutics18010013

**Published:** 2025-12-22

**Authors:** Valeria Carini, Giulia Scagnetti, Joanne Foulkes, Katie Evans, Imran Saleem, Sarah Gordon

**Affiliations:** 1 School of Pharmacy and Biomolecular Sciences, Liverpool John Moores University, Liverpool L3 3AF, UK; valeriacarini.92@gmail.com (V.C.); giulia.scagnetti@gmail.com (G.S.); j.m.foulkes@ljmu.ac.uk (J.F.); k.evans@ljmu.ac.uk (K.E.); i.saleem@ljmu.ac.uk (I.S.); 2 School of Pharmacy, School of Health Sciences, College of Medicine and Health, University of Birmingham, Birmingham B15 2TT, UK

**Keywords:** lipid-polymer hybrid nanoparticle, bacteriomimetic, antimicrobial peptide, anti-infective, co-delivery, combination delivery, microfluidic manufacture

## Abstract

**Background/Objectives:** The growing threat posed by antimicrobial resistance to worldwide public health highlights the urgent need not only for new anti-infective candidates, but also for innovative formulation strategies capable of mediating effective delivery of anti-infective compounds. The current study, therefore, aimed to demonstrate the feasibility of formulating lipid-polymer hybrid nanoparticles (LPHNPs) with dual loading of both core and shell compartments for combination anti-infective delivery. **Methods:** LPHNPs containing the antibiotic cefotaxime within a chitosan polymer core and the novel antimicrobial peptide RN7IN6 within a bacteria-mimicking lipid shell were produced by microfluidic mixing, and optimized with respect to parameters including total flow rate, flow rate ratio, and lipid concentration. Minimum inhibitory concentrations of cefotaxime and RN7IN6 co-incorporated in LPHNPs were assessed as a preliminary indicator of antibacterial efficacy. **Results:** Uniformly nanosized LPHNPs were produced, with maximized loading of cefotaxime and RN7IN6 within particle cores and shells, respectively. Empty LPHNPs showed an appreciable antibacterial activity, particularly against the Gram-negative bacterium *Escherichia coli*, while RN7IN6 was indicated to enhance cefotaxime activity against *E. coli* when both actives were incorporated in LPHNPs. **Conclusions:** The current findings clearly demonstrate the feasibility of formulating LPHNPs for core-shell co-encapsulation and delivery of anti-infectives. The promising antibacterial efficacy of co-loaded LPHNPs warrants further in-depth investigation to determine the extent of co-loaded LPHNP applications as combination anti-infective delivery platforms.

## 1. Introduction

Antimicrobial resistance (AMR) is a major and growing threat to public health worldwide, with significant effects on global morbidity, mortality, and healthcare costs. Unless impactful measures can be implemented, drug-resistant infections have been projected to result in 10 million deaths globally by 2050 [1]. Bacterial AMR, of particular concern, has already been reported to have contributed to 4.95 million deaths worldwide in 2019 alone [2], with bacterial cell envelope structures often acting as significant barriers to effective anti-infective delivery even in wild-type organisms [3]. As a further problem, diminishing flow through the antibiotic pipeline means that the shrinking pool of effective antibiotics is not being refilled by additional or novel treatment options [4,5]. This escalating global crisis draws attention to an urgent need not only for new anti-infective candidates with alternative targets and/or modes of action to traditional antibiotics, but also importantly, for innovative strategies to facilitate the effective delivery of novel and traditional anti-infective therapies into and across bacterial cell envelope structures to reach their targets.

Lipid-polymer hybrid nanoparticles (LPHNPs) consisting of a robust polymer core surrounded by a membrane-like lipid shell have been widely investigated to date for the delivery of cancer therapeutics [6,7,8], and are emerging as a delivery strategy of promise in the context of infection research. LPHNPs combine the benefits (and may circumvent the limitations) of polymeric nanoparticles and liposomes as separate delivery systems—the LPHNP polymeric core provides a high structural stability and capacity for drug loading, while the lipid shell confers an enhanced biocompatibility, and the potential to prevent premature escape or even affect controlled release of drug payload [9,10]. In an innovative step, the lipid shell component can be further engineered to consist of native bacterial cell membranes or synthetic bacteria-relevant lipid mixtures, leading to the fabrication of bacteriomimetic LPHNPs possessing the potential for enhanced interaction with and improved anti-infective delivery to target bacterial cell envelopes [11,12,13].

The distinct two-compartment structure of LPHNPs further creates the possibility for co-encapsulation of hydrophilic and lipophilic actives within a single carrier structure. While core-shell loading of LPHNPs with chemotherapeutic agents has been previously trialed [8,14], to the best of the authors’ knowledge, incorporation of anti-infectives separately into core and shell structures of LPHNPs has not been extensively investigated. This is of significant interest in the context of combination therapy, which involves the simultaneous use of anti-infectives of differing but complementary modes of action. Delivering combinations of anti-infectives may lead to an enhanced antibacterial activity compared to single-agent use, in turn facilitating a reduction in therapeutic drug dose and mitigating resistance development [15,16]. The considerable potential for individual actives within a combination to have differing physicochemical properties (including water solubility/lipophilicity), however, creates practical challenges for co-administration, and as such may present a hurdle to effective translation of certain combination approaches. This renders the ability to incorporate and deliver combinations of anti-infectives via a single nanocarrier platform an attractive prospect.

The current work was, therefore, designed to serve as an in-depth proof-of-concept study, with the aim of exploring the feasibility of formulating LPHNPs as dual-compartment platforms for co-encapsulation and delivery of anti-infective compounds. To this end, a process for the production of LPHNPs consisting of a chitosan polymer core encapsulating the traditional antibiotic cefotaxime and a bacteria-relevant lipid shell incorporating the novel antimicrobial peptide RN7IN6 was established. With a view to creating clear possibilities for scale-up and translation, a microfluidic mixing technique was employed for this purpose, and a detailed investigation of the impact of varying parameters such as flow rate ratio (FRR), total flow rate (TFR), and component concentrations was conducted. This process was optimized to produce uniformly nanosized LPHNPs, with maximal anti-infective co-loading. Co-loaded LPHNPs were then screened in a preliminary antibacterial efficacy study to gauge the extent of their future promise as combination anti-infective delivery platforms. The study demonstrates a considerable potential for co-loaded LPHNPs as platforms for combination anti-infective delivery, and highlights a number of key focal points for further, in-depth investigation to fully explore the extent of this potential.

## 2. Materials and Methods

### 2.1. Materials

Mueller–Hinton Broth 2 (MHB2), nutrient agar, and resazurin were purchased from Sigma-Aldrich (St. Louis, MO, USA). Chitosan hydrochloride (MW 30–400 kDa, degree of deacetylation 80–95%) was obtained from Heppe Medical Chitosan GmbH (Halle, Germany). Phosphate-buffered saline (PBS, pH 7.4) tablets were obtained from Oxoid Ltd. (Basingstoke, UK). 1-palmitoyl-2-oleoyl-sn-glycero-3-phosphoethanolamine (POPE), 1-palmitoyl-2-oleoyl-sn-glycero-3-phospho-(1′-rac-glycerol) (sodium salt) (POPG), and 1′,3′-bis [1,2-dioleoyl-sn-glycero-3-phospho]- glycerol (sodium salt) (tetraoleoyl cardiolipin, CL) were purchased from Avanti Polar Lipids (Merck, Rahway, NJ, USA). HPLC-grade acetonitrile and methanol were purchased from Thermo Fisher Scientific (Waltham, MA, USA). N,N-dimethylformamide (DMF) was bought from Acros Organics (Bridgewater, NJ, USA). 1-propanol and formic acid were purchased from VWR (Radnor, PA, USA). Trifluoroacetic acid (TFA) was purchased from Fisher Scientific (Hampton, NH, USA). Cefotaxime sodium salt (CTX, ≥95%) was obtained from Enzo Life Sciences (Exeter, UK). The antimicrobial peptide (AMP) RN7IN6 (amino acid sequence FLGGLIKWWPWRR-NH_2_, MW 1709.078 g/mol [17] purity > 95%) was synthesized and purified in-house [18], as detailed in the Supplementary Materials.

### 2.2. Empty LPHNP Manufacture and Characterization

Empty LPHNPs consisting of a chitosan nanoparticle (CNP) core surrounded by a bacteria-relevant lipid shell (POPE, POPG, and CL in a 70:20:10 wt% mixture corresponding to a 15:4:1 molar ratio, representative of the Gram-negative bacterial inner membrane [19]) were manufactured by microfluidic mixing via a two-step method. In the first step, a NanoAssemblr^®^ Benchtop system equipped with a staggered herringbone mixer cartridge (Precision Nanosystems Inc., Vancouver, BC, Canada, cartridge as illustrated in [20]) was used to manufacture empty CNPs (see Supplementary Materials Section S1.1 for empty CNP preparation and optimization). As a second step, CNP were coated with bacteria-relevant lipids to produce empty LPHNP by injecting unpurified and undiluted CNP into one inlet of the micromixer, and a POPE:POPG:CL lipid solution (prepared by dissolving lipids in 1-propanol:DMF (80:20 *v*/*v*) containing 0.01% formic acid) into the other inlet, at room temperature.

Two screening studies were initially conducted, which allowed for the determination of optimal lipid concentration and TFR parameters for empty LPHNP production (see Supplementary Materials Section S1.3). In order to determine the optimal FRR (CNP dispersion:lipid solution), empty LPHNPs were prepared using a set optimized lipid concentration of 0.5 mg/mL and a TFR of 20 mL/min, and FRR values ranging from 2:1 to 6:1 (Table 1).

### 2.3. CTX-Loaded LPHNP Manufacture and Characterization

CTX-loaded LPHNPs were formulated as a CTX-loaded CNP core (see Supplementary Materials Section S1.2 for CTX-loaded CNP preparation and optimization, with 3 mg/mL CTX utilized as optimal loading concentration), surrounded by a bacteria-relevant lipid shell as described in Section 2.2. CTX-loaded LPHNPs were prepared by microfluidic mixing of CTX-loaded CNP and lipid solutions similar to that described for empty LPHNPs in Section 2.2, and employing optimized lipid concentration, TFR, and FRR parameters as established for empty LPHNPs.

### 2.4. CTX and RN7IN6 Co-Loaded LPHNP Manufacturing and Characterization

The microfluidic manufacturing process and parameters used to prepare CTX (polymer core) and RN7IN6 (lipid shell) co-loaded LPHNPs were as described and optimized in Section 2.2 and Section 2.3. To investigate the additional optimal loading of RN7IN6 into LPHNP lipid shells, increasing loading concentrations of RN7IN6 ranging from 0.5 mg/mL to 2 mg/mL were dissolved in the organic phase together with lipid shell constituents, as summarized in Table 2.

### 2.5. Physical Characterization of Nanoparticles

The z-average (mean hydrodynamic diameter), PDI, and zeta potential as critical quality attributes of CNPs and LPHNPs were measured via dynamic light scattering/electrophoretic mobility, using a Zetasizer Nano ZS (Malvern Instruments Ltd., Malvern, UK) following appropriate dilution of samples in PBS (pH 7.4), and at a temperature of 25 °C.

### 2.6. Nanoparticle Purification

Residual organic solvent and unentrapped actives (as applicable) were removed from formulations using Centrisart^®^ centrifugal ultrafiltration units (300 kDa molecular weight cutoff, Sartorius, Epsom, UK). A 2 mL volume of nanoparticle sample was loaded into centrifugal ultrafiltration units and centrifuged at 1188× *g* and 25 °C for 30 min (Eppendorf centrifuge 5804r, Hamburg, Germany). Concentrated LPHNP dispersions were then collected and diluted for further use as required. Where appropriate, the produced ultrafiltrate was used for the determination of encapsulation efficiency (EE%) and incorporated concentration of CTX and/or RN7IN6 (Section 2.7).

### 2.7. Quantification of CTX and RN7IN6

An HPLC system (1200 series) from Agilent Technologies (Santa Clara, CA, USA) was used for quantification of CTX and RN7IN6. The system was operated via ChemStation software (version LTS 01.11) and was equipped with a G1322A degasser, a G1312A binary pump, a G1316A thermostated column compartment, a G1329A autosampler, and a G1314B variable wavelength detector. The analysis method was employed to quantify both CTX and RN7IN6, with chromatographic separation carried out by employing an Agilent Eclipse XDB-C18 column (4.6 × 150 mm, particle size 5 μm, Santa Clara, CA, USA) and measuring UV absorbance at 215 nm. The mobile phase consisted of eluent A (ultrapure water/acetonitrile 95/5%, 0.1% TFA) and eluent B (ultrapure water/acetonitrile 5/95%, 0.1% TFA). Samples were run with a gradient of 100% to 0% eluent A over 20 min at 0.8 mL/min and 25 °C (injection volume 10 μL). Following each sample run, the system was flushed with 100% eluent B for 3 min and re-equilibrated for 11 min. Data were analyzed with reference to CTX and RN7IN6 calibration curves.

### 2.8. Evaluation of CTX and RN7IN6 EE% and Incorporated Drug Concentration

The EE% and incorporated drug concentrations of CTX and RN7IN6 were determined indirectly by HPLC-based quantification of unentrapped compound (see Section 2.7) in ultrafiltrate samples resulting from centrifugal ultrafiltration of LPHNP formulations as described in Section 2.6. EE% was then calculated by inferring the entrapped compound amount and expressing this as a percentage of the theoretical amount of active added during formulation preparation. The inferred amount of entrapped compound was also used to calculate an incorporated drug concentration, expressed as an amount of CTX or RN7IN6 contained within 1 mL of LPHNP formulation (μg/mL).

### 2.9. CTX and RN7IN6 Release Kinetics

Release profiles of CTX from CTX-loaded CNPs, CTX-loaded LPHNPs, and CTX and RN7IN6 from co-loaded LPHNPs were determined. Purified formulations were diluted in 9 mL of PBS (pH 7.4) to give initial concentrations of approx. 100 μg/mL CTX and 39 μg/mL RN7IN6. The samples were then placed on a HulaMixer^™^ Sample Mixer (Thermo Fisher Scientific, Waltham, MA, USA) and continuously rotated at a temperature of 37 °C. At specific timepoints (0, 1, 2, 4, 6, 24, and 48 h), 500 μL of supernatant produced by ultracentrifugation (55,000 rpm, 1 h, 4 °C, rotor type 70.1 Ti, Beckman Coulter Inc., Brea, CA, USA) was withdrawn and replaced with an equal volume of fresh PBS. Release of CTX or RN7IN6 (cumulative percentage) was calculated following HPLC analysis of supernatant samples as described in Section 2.7.

### 2.10. Antimicrobial Efficacy Assessment

#### 2.10.1. Bacteria

Free CTX and RN7IN6 and actives incorporated in various CNP and LPHNP formulations were tested for antimicrobial activity against *Escherichia coli* (NCTC 12241) and *Staphylococcus aureus* (NCTC 12981) (National Collection of Type Cultures, Salisbury, UK).

For all antimicrobial testing, preparation of a standardized bacterial suspension was achieved by suspending a single bacterial colony from an overnight culture on nutrient agar in 50 mL of MHB2, which was incubated overnight at 37 °C in an orbital shaking incubator at 250 rpm. A 40 μL volume of the overnight culture was then used to inoculate 150 mL of MHB2, which was incubated at 37 °C for 3 h in an orbital shaking incubator. Bacterial suspensions were adjusted to OD_600_ 0.05–0.1, corresponding to 1 × 10^6^ CFU/mL (as determined from bacterial growth curves), following OD_600_ measurement on a SpectroStar Nano microplate reader (BMG LABTECH, Ortenberg, Germany).

#### 2.10.2. Free CTX and RN7IN6 Antibacterial Activity

The antibacterial activity of free CTX and free RN7IN6 alone was evaluated by a microdilution method incorporating resazurin [21] to determine the minimum inhibitory concentration (MIC). Experiments were performed in 96-well plates, in triplicate. Bacterial suspensions were prepared as in Section 2.10.1 and diluted in 96-well plates to a final concentration per well of 5 × 10^5^ CFU/mL. Doubling dilutions of anti-infectives were added into 96-well plates to reach a final volume per well of 200 μL. Plates were incubated at 37 °C overnight. The MIC endpoint was visually evaluated as the lowest concentration of CTX or RN7IN6, which prevented a color change from blue to pink. Negative (MHB2 + resazurin solution) and positive (MHB2 + resazurin solution + inoculum) controls were also included, with additional controls (MHB2 + resazurin + RN7IN6 solution, and MHB2 + resazurin + CTX solution) to verify that RN7IN6 and CTX did not affect the turbidity or color of the broth.

#### 2.10.3. CTX and RN7IN6 Synergism Assessment (Microdilution Checkerboard Assay)

To determine whether co-administration of CTX and RN7IN6 could lead to an enhanced antibacterial effect, synergy analysis was carried out by a checkerboard titration method using 96-well microtiter plates [22]. Concentration ranges of CTX and RN7IN6 employed in the checkerboard assay (CTX: 16–0.25 μg/mL for *S. aureus*, 1–0.016 μg/mL for *E. coli*; RN7IN6: 128–2 μg/mL for *S. aureus*, 256–4 μg/mL for *E. coli*) were selected according to free CTX and RN7IN6 MIC values.

An 80 μL volume of standardized bacterial suspension (see Section 2.10.1) was added to each plate well. CTX and RN7IN6 were then added in 2-fold serial dilutions down columns (CTX) and across rows (RN7IN6) of microtiter plates. Control columns and wells of doubly diluted CTX or RN7IN6 alone, respectively, were also plated. Following dilution of antimicrobial agents, 20 μL of sterile resazurin solution in MHB2 (0.2 mg/mL) was added to the plate. Negative controls (MHB2 + resazurin solution, MHB2 + resazurin + RN7IN6 solution, or CTX solution) and positive controls (MHB2 + resazurin solution + inoculum) were performed alongside each experiment. MIC values for CTX and RN7IN6, both alone and in combination, were visually evaluated as described in 2.10.2.

Determined MICs were then used to calculate a fractional inhibitory concentration index (FICI) to assess the interaction between CTX and RN7IN6 in both *S. aureus* and *E. coli*. The fractional inhibitory concentration (FIC) of CTX and of RN7IN6 was first calculated, as the fraction of the MIC of each active in combination relative to its MIC alone; the FICI was then calculated as the sum of CTX and RN7IN6 FIC values, and interpreted as follows [23]:

- FICI < 0.5: synergistic activity;
- 0.5 < FICI < 1: partial synergism;
- FICI = 1: addition;
- 1 < FICI < 4: indifference;
- FICI ≥ 4: antagonism.

#### 2.10.4. Antimicrobial Activity of Empty and Loaded LPHNPs

The antibacterial activity of empty, CTX-loaded, and co-loaded LPHNPs, as well as CTX-loaded CNPs, was assessed as described in Section 2.10.2. All formulations were first purified according to Section 2.6, then diluted in sterile PBS (pH 7.4) to reach desired incorporated concentrations of CTX, or CTX and RN7IN6 in combination, as follows:

- CTX-CNPs and CTX-LPHNPs were diluted to reach a CTX concentration of 512 μg/mL for *S. aureus* and 16 μg/mL for *E. coli*;
- Co-loaded LPHNPs were diluted based on RN7IN6 content to 32× MIC of free RN7IN6, corresponding to 512 μg/mL for *S. aureus* and 1024 μg/mL for *E. coli.* The corresponding CTX concentration was 765 μg/mL and 1530 μg/mL for *S. aureus* and *E. coli*, respectively;
- Empty LPHNPs were diluted in the same manner as for CTX-CNPs and CTX-LPHNPs.

### 2.11. Statistical Analysis

Statistical analysis of the physical characteristics of empty and CTX-loaded LPHNPs was performed using IBM SPSS Statistics software (Version 30.0.0.0, IBM Corp., Armonk, NY, USA). One-way ANOVA with Tukey’s post hoc test was performed to assess mean differences in LPHNP size, PDI, and zeta potential. A *p* value of <0.05 was taken as indicating statistical significance.

Statistical analysis of co-loaded LPHNPs was performed using JMP^®^ 16.2.0 software (SAS Institute Inc., Cary, NC, USA). An independent *t*-test was performed on co-loaded LPHNP size, PDI, and Z-potential measurements to determine if there was a mean difference in formulation physicochemical characteristics before and after purification via centrifugation ultrafiltration. If the output of Levene’s test to assess equality of variance was *p* > 0.05, the two-sided *p* value with equal variance assumed (pooled *t*-test) was considered, while if the output of Levene’s test was *p* < 0.05, the two-sided *p* value with equal variance not assumed (unpooled *t*-test) was considered. A *p*-value of <0.05 was taken as indicating statistical significance throughout the study. Further, a one-way ANOVA with Tukey’s post hoc test was employed to compare the physicochemical characteristics of co-loaded LPHNPs and determine any significant differences resulting from varying RN7IN6 loading concentration. A *p* value of <0.05 was taken as indicating statistical significance.

## 3. Results and Discussion

### 3.1. Empty LPHNP Manufacture and Characterization

The current work aimed to investigate the unexplored potential of LPHNPs as dual-compartment platforms for combination anti-infective delivery. As an initial investigative step, the feasibility of this approach from a formulation perspective was explored in this study. Mixed methodologies employing variations of thin film hydration [24,25,26,27] or vesicle fusion [28,29] have traditionally been used for LPHNP formulation; while entirely suitable for small batch production, such methods present challenges for scalable LPHNP manufacture [30]. A microfluidic mixing-based approach was therefore employed within this proof-of-concept study, as a scalable alternative offering precise control over production parameters. With future in-depth investigation of their antibacterial applications in mind, LPHNPs were further designed with a Gram-negative bacterial membrane-mimicking POPE:POPG:CL lipid shell [31] as a strategy hypothesized to enhance interaction with target bacterial membranes [12,32,33].

**Figure 1:**
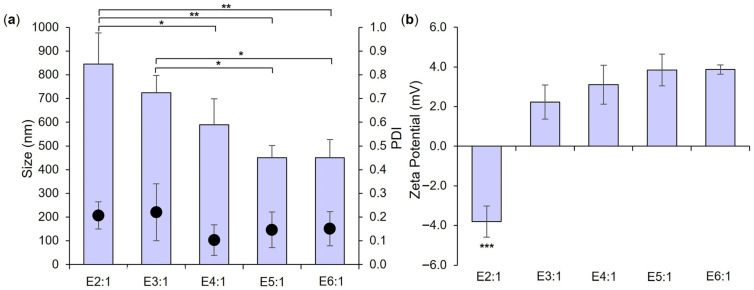
Size and PDI (bars and dots, respectively, (**a**)) and zeta potential (**b**) of empty LPHNP formulations prepared using a lipid concentration of 0.5 mg/mL, a TFR of 20 mL/min, and FRR as indicated in formulation names and in Table 1. Results represent mean ± SD of *n* = 3. * = *p* < 0.05 and ** = *p* < 0.01 with respect to formulations indicated in (**a**), and *** = *p* < 0.001 for E2:1 relative to all other formulations in (**b**).

A progressive development process for the production of co-loaded LPHNPs was employed, with empty LPHNP production serving as the first stage in this process. Empty LPHNPs were manufactured using a rapid two-step microfluidic process in which CNP as polymer cores were first produced (optimized as detailed in the Supplementary Materials), followed by the input of CNP and bacteria-relevant lipid solution into separate channels of the micromixer to achieve controlled mixing and coating of polymer cores with a lipid shell. As key manufacturing parameters in this second coating step, the impact of varying lipid concentration, TFR, and FRR (CNPs:lipid solution) on empty LPHNP size, PDI, and zeta potential was investigated. Initial scoping studies employing Taguchi (orthogonal array, Figure S4) followed by full factorial design of experiments (Figure S5) identified the lowest employed lipid concentration (0.5 mg/mL) and a TFR of 20 mL/min as conditions resulting particularly in a small and uniform LPHNP size. These parameters were then fixed, and further investigation was conducted employing varying FRR values (Table 1) to allow for focus on identification of the optimal value for this manufacturing parameter (Figure 1).

**Figure 2:**
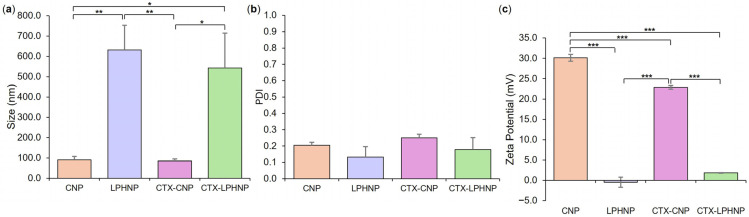
Size (**a**), PDI (**b**), and zeta potential (**c**) of empty and CTX-loaded CNP and LPHNP formulations. Results represent mean ± SD of *n* = 3. * = *p* < 0.05, ** = *p* < 0.01, and *** = *p* < 0.001 with respect to formulations indicated.

As anticipated from Figure S5 and published literature, increasing the FRR of CNP to lipid solution resulted in a trend toward a smaller empty LPHNP size (Figure 1a) and a change in surface charge nature (Figure 1b), with significant differences noted in both parameters between formulations employing the lowest (E2:1) and highest (E6:1) FRR values. Polymer/lipid ratios have been reported to play a key role in controlling LPHNP size and surface charge—too low a ratio may potentially lead to the formation of lipid-component liposomes, resulting in an overall increase in measured size and a (more negative) liposome-approximating zeta potential, while too high a ratio may result in incomplete lipid coating of polymer cores and measurement of a (highly positive, in this case) polymer core-approximating zeta potential [34]. Neither observation is evident in Figure 1. While no significant difference in size, PDI, or zeta potential was noted between formulations E4:1, E5:1, and E6:1 employing the highest FRR investigated, the 5:1 FRR of E5:1 was selected for further use due to a trend toward a smaller particle size in comparison to E4:1, and the greater lipid concentration employed relative to E6:1 (which may potentially facilitate a greater LPHNP lipid shell loading capacity). Further investigation confirmed that employing a total lipid concentration of 0.5 mg/mL, TFR of 20 mL/min, and FRR of 5:1 resulted in the successful formation of a lipid shell around polymer cores. A significant increase in the size of empty LPHNPs relative to empty CNP cores was noted, as well as a significant reduction in zeta potential, indicating effective coverage and masking of cationic CNP surfaces with a negatively charged lipid shell (POPE = zwitterionic, POPG and CL = anionic) (Figure 2) [34,35].

### 3.2. Fabrication and Characterization of CTX-Loaded LPHNPs

As a next step toward the production of a co-loaded formulation, LPHNPs with drug-loaded polymeric cores were produced and optimized. This was achieved by first maximizing the loading of the traditional antibiotic CTX into CNP polymer core structures (see Supplementary Material), followed by the employment of lipid concentration, TFR, and FRR parameters established in the context of empty LPHNP formulation, in order to envelop CTX-loaded CNP cores within a bacteria-relevant lipid shell. Investigation and comparison of formulation critical quality attributes showed that CTX-loaded LPHNPs were significantly larger (Figure 2a) with a lower zeta potential (Figure 2c) than CTX-loaded CNP. This was entirely consistent with empty LPHNP vs. CNP findings and further indicated a lack of impact of CTX incorporation on the ability to successfully coat polymer cores with a lipid shell.

When comparing polymer core structures, no significant difference was noted in the size and PDI of empty vs. CTX-loaded CNPs; however, a slight but significant reduction in CNP zeta potential was noted as a result of drug loading (30.1 ± 0.8 mV for empty vs. 22.9 ± 1.0 mV for CTX-CNP, Figure 2). This is not unexpected, with previous studies having also noted the ability of zwitterionic cefotaxime to reduce the magnitude of nanoparticle zeta potential, likely due to a degree of drug surface adsorption in addition to encapsulation [36]. This effect did not translate to the LPHNP level, with no significant difference in zeta potential (or size distribution) between empty and CTX-loaded LPHNPs, indicating that the physicochemical characteristics of LPHNPs remained consistent regardless of CTX incorporation into polymer cores [37].

### 3.3. Co-Loaded LPHNP Production: Optimization of RN7IN6 Loading Concentration

Following successful optimization of empty LPHNP followed by CTX-loaded LPHNP production, incorporation of RN7IN6 within lipid shells was investigated as the final step in the manufacture of co-loaded LPHNPs. As a novel, synthetic hybrid of the naturally occurring AMPs indolicidin and ranalexin, RN7IN6 was utilized due to its promising (but not thoroughly investigated) antibacterial activity, facilitated by bacterial membrane disruption and permeabilization [17,38]. RN7IN6 was specifically incorporated within the lipid shell compartment of LPHNPs due to its amphiphilic nature [17], and as a future-focused strategy to achieve efficient localization of the AMP to bacterial membrane target sites upon LPHNP lipid shell–bacterial membrane interactions.

**Figure 3:**
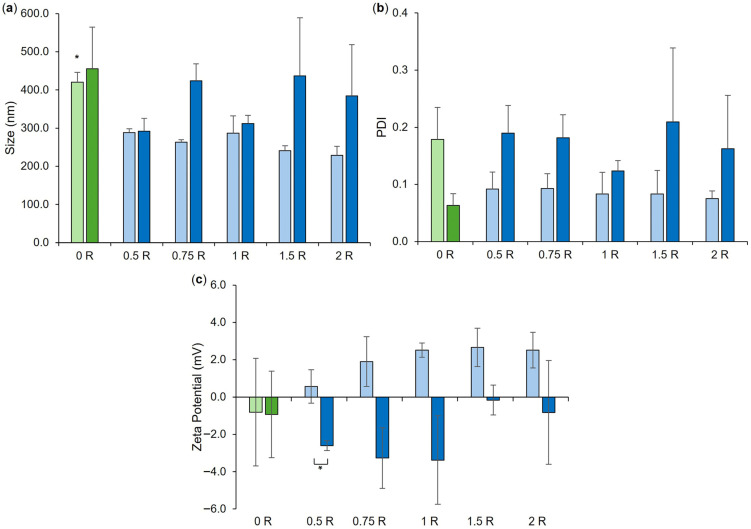
Size (**a**), PDI (**b**), and zeta potential (**c**) of co-loaded LPHNP formulations, loaded with 0.5–2 mg/mL RN7IN6 as specified in Table 2 and in formulation names. ‘0 R’ represents CTX-LPHNP with no incorporated RN7IN6. Lighter shades of green and blue designate formulations after preparation, and darker shades indicate measurement after formulation purification. Results represent mean ± SD of *n* = 3. * = *p* < 0.05 for the size of CTX-LPHNPs (‘0 R’) relative to all other formulations after preparation in (**a**) and for formulations as indicated in (**c**).

**Figure 4:**
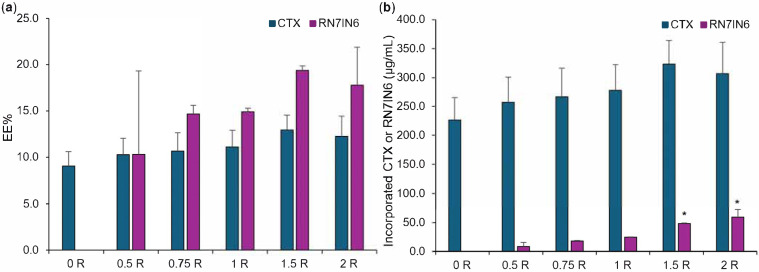
CTX and RN7IN6 encapsulation efficiency (EE%, (**a**)) and incorporated concentration (**b**) determined in co-loaded LPHNPs prepared using 0.5–2 mg/mL RN7IN6, as specified in Table 2 and in formulation names. ‘0 R’ represents CTX-LPHNP with no incorporated RN7IN6. Results represent mean ± SD of *n* = 3. * = *p* < 0.05 with respect to the concentration of incorporated RN7IN6 of formulations loaded with 1.5 mg/mL (‘1.5 R’) and 2 mg/mL (‘2 R’) of RN7IN6, in comparison to all other formulations loaded with 0–1 mg/mL RN7IN6 (**b**).

LPHNPs with polymer core-loaded CTX were further optimized with a view to achieving maximal RN7IN6 encapsulation within the lipid shell, while maintaining a small and uniform LPHNP size distribution. With previously established formulation parameters fixed, the impact of increasing RN7IN6 loading concentrations on LPHNP size, PDI, and zeta potential (Figure 3) as well as CTX/RN7IN6 EE% and incorporated concentrations (Figure 4) was investigated. In careful consideration of the potential for purification processes to induce particle fusion or aggregation [39,40], in addition to the potential risks to colloidal stability indicated by the near-neutral zeta potential [32,35] of LPHNPs prior to AMP loading of lipid shells, the physical characteristics of co-loaded LPHNPs were examined both immediately after preparation and following purification by centrifugal ultrafiltration (Figure 3).

A minimal impact of centrifugal ultrafiltration was noted on co-loaded LPHNP physical properties, with statistical analysis demonstrating no difference in LPHNP size and PDI pre- and post-purification (Figure 3a,b), and only co-loaded LPHNPs produced by loading with 0.5 mg/mL RN7IN6 showed a slight but significant difference in surface charge magnitude following purification (Figure 3c). Comparative analysis performed on co-loaded LPHNPs to assess the impact of RN7IN6 loading and concentration effects (illustrated where applicable in Figure 3, elaborated on in Figure S6) showed a significant difference in the size of all co-loaded LPHNPs independent of loading concentration in comparison to CTX-loaded LPHNPs, prior to purification (Figure 3a); however, this was anticipated to be an artifact of free/unencapsulated peptide [41,42] as no significant difference in LPHNP size with incorporation of RN7IN6 at any concentration was noted following purification. Interestingly, while an overall trend toward more positive zeta potential values was noted with increasing RN7IN6 loading concentrations in both pre- and post-purification data (Figure 3c), this was not significant in either case. A more notable shift toward positive zeta potential values with incorporation of RN7IN6 into LPHNP lipid shells was anticipated, given the highly positive net charge of RN7IN6 [17] and expected localization of hydrophilic AMP residues at LPHNP surfaces. This may not have occurred due to the documented potential for interactions between cationic and aromatic sidechains of amino acids, such as those present in RN7IN6, known to result in shielding of cationic residues and deeper insertion of peptide into lipid membrane structures [38,43].

The EE% and incorporated concentration of CTX were comparable across all LPHNP formulations (Figure 4), which was expected given that all LPHNPs were manufactured by coating of polymer core structures prepared using the same CTX loading concentration. This observation further demonstrates that additional loading of LPHNP with RN7IN6 did not impact CTX incorporation, with statistical analysis confirming no significant differences in CTX EE% or incorporated concentration between CTX-only and co-loaded LPHNPs (see Figure S7).

RN7IN6 showed a trend for increasing EE% with loading concentration, with values increasing from 10.3% to 17.8% (Figure 4a); however, this trend was not statistically significant. In contrast, the concentration of RN7IN6 incorporated into co-loaded LPHNPs was observed to increase in a statistically significant manner over the loading concentration range employed (illustrated in Figure 4b, elaborated on in Figure S7), demonstrating the importance of an in-depth approach to physicochemical characterization during the LPHNP optimization process. It would be of future interest to determine whether lipid shell loading capacity was saturated with the highest loading concentrations employed in the current work, or even greater concentrations of incorporated RN7IN6 could be achieved.

Given the similarity in physical characteristics, CTX loading, and RN7IN6 EE% for all investigated co-loaded LPHNP formulations, the maximal observed concentration of incorporated RN7IN6 was used as the sole decision criterion for optimal RN7IN6 loading concentration. As such, a 2 mg/mL loading concentration of RN7IN6 was determined as optimal and was employed for all further co-loaded LPHNP formulation manufacture.

### 3.4. CTX and RN7IN6 Release Kinetics

**Figure 5:**
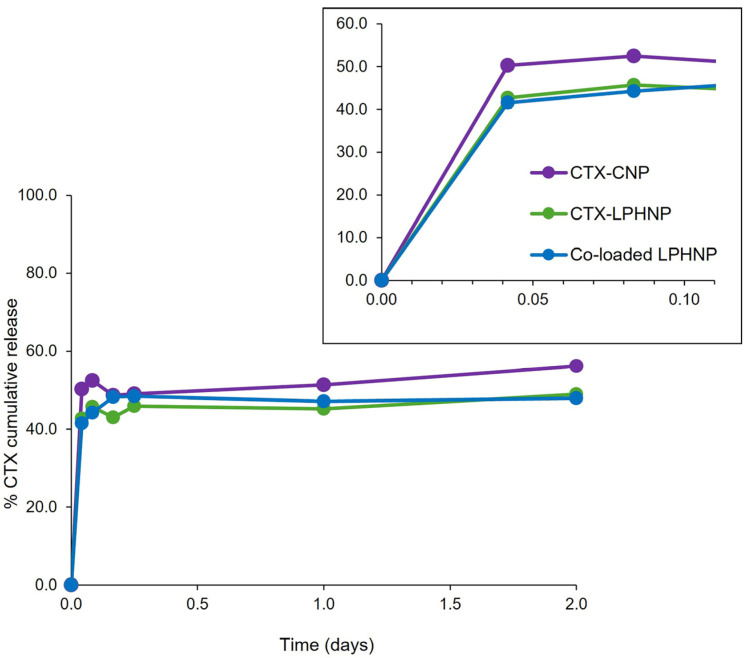
Percentage cumulative release of CTX from CTX-CNP, CTX-LPHNP and co-loaded LPHNP, with inset illustrating burst release within approximately the first two hours. Release of RN7IN6 from co-loaded LPHNPs could not be detected. Results represent mean ± SD of triplicate measurements.

Following the successful establishment of optimized parameters for the preparation of co-loaded LPHNPs, the release kinetics of CTX and RN7IN6 from the co-loaded formulation were investigated. Release of CTX from CTX-loaded CNP and CTX-loaded LPHNP was also assessed, to allow for the determination of the impact of the LPHNP lipid shell and further incorporation of RN7IN6 on CTX release (Figure 5).

**Figure 6:**
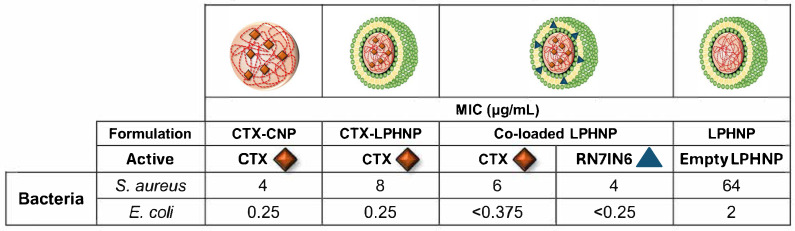
Minimum inhibitory concentration (MIC) values of CTX in CTX-CNP, CTX-LPHNP, and co-loaded LPHNP, and RN7IN6 in co-loaded LPHNP, against *S. aureus* and *E. coli*. Red diamonds indicate CTX, while blue triangles denote RN7IN6, with the location of active encapsulation within CNP or LPHNP as illustrated in images. The MIC value of empty LPHNPs (diluted by the same factor as CTX-loaded CNP and LPHNP formulations) is also shown. Mean MIC values of triplicate results are shown in each case—replicates yielded identical inhibitory concentrations and therefore values are reported as measured, without rounding or SD.

A 48-hour time course was examined to approximate the duration of subsequent antimicrobial activity assays (see Figure 6) and allow for specific characterization of release behavior relevant to the timeframe of this preliminary efficacy assessment. Figure 5 shows only CTX release from various formulations, as the release of RN7IN6 from co-loaded LPHNPs could not be detected. This is not entirely surprising given the affinity of AMPs such as RN7IN6 for bacterial membrane structures [44,45] and the previously discussed potential for RN7IN6 to migrate into LPHNP lipid shells [38,43]. Indication of preserved antimicrobial activity of RN7IN6 in co-loaded LPHNPs (see Figure 6) would support the hypothesis that no release of RN7IN6 was detected from co-loaded LPHNPs due to a strong AMP affinity for the LPHNP lipid shell, rather than as a result of peptide instability or inactivation; this observation also adds weight to the suggestion that the bacteria-relevant LPHNP shell composition may promote interaction with bacterial membrane structures and, in doing so, allow for direct transfer of AMP cargo into bacterial cell envelopes [12,33].

Similar CTX release profiles were noted across investigated nanoparticle formulations, with an initial burst release occurring within the first 2 h (see inset, Figure 5), followed by a much slower, sustained release. The presence of RN7IN6 in co-loaded lipid shells was, therefore, not deemed to have an appreciable impact on CTX release. While a slightly greater burst release of CTX may be noted from CNPs in comparison to LPHNP formulations (see inset, Figure 5), the lipid shell of LPHNPs appears to have affected only a modest reduction in this initial burst and a minor impact on the overall CTX release profile. This is in contrast to the literature, which has reported a considerable impact of LPHNP lipid shells on drug release kinetics [46,47]. This discrepancy may be a result of the novel, bacteria-relevant lipid shell components of the currently investigated LPHNP formulations. The addition of POPG to POPE membrane structures has been reported to lead to an increase in membrane fluidity [48], while the inclusion of CL at molar concentrations as employed in the current study has also been shown to induce flexibility within membrane structures [49]. Such fluidizing effects are beneficial with respect to their potential to enhance LPHNP–bacteria interactions, and may also render the POPE:POPG:CL lipid shell a relatively permeable structure to CTX released from LPHNP polymer cores.

### 3.5. Antibacterial Efficacy of CTX and RN7IN6 in Combination

While the major focus of this study was on demonstrating the feasibility of formulating dual-compartment loaded LPHNPs, having established a uniformly nanosized co-loaded formulation with maximal CTX and RN7IN6 loading, it was of interest to preliminarily investigate the antimicrobial impact of this formulation to provide experimental direction for future work. Given the novelty of this particular anti-infective combination, baseline MIC values for free CTX and RN7IN6 alone, followed by their MICs when employed in combination, were investigated prior to employing LPHNPs, in order to assess their level of synergism (Table 3). *S. aureus* and *E. coli* were employed as target organisms for antibacterial testing, as examples of Gram-positive and Gram-negative bacterial pathogens, respectively, of high public health importance [50].

For both *S. aureus* and *E. coli*, a combination of RN7IN6 and CTX resulted in a partial synergistic effect, with 0.5 < FICI < 1 (Table 3). This reflects a moderate but notable potentiation of antimicrobial activity when the two agents are combined, and interestingly demonstrates the ability of a novel AMP to enhance the antibacterial activity of a traditional antibiotic against both Gram-positive and Gram-negative pathogens. This is in agreement with published work, which has previously shown that membranolytic peptides can alter the shape of bacterial cells by creating pores, improving access of β-lactam antibiotics such as CTX to their targets, and allowing them to inflict significant damage on bacterial cell walls [51,52].

### 3.6. Co-Loaded LPHNPs: Preliminary Assessment of Antibacterial Efficacy

The observed ability of RN7IN6 to potentiate the activity of CTX was then further investigated with both compounds co-incorporated in LPHNPs. This perspective was adopted rather than focusing on probing the occurrence of synergism due to the intention for this investigation to serve as a preliminary indicator of LPHNP antibacterial impact to direct further work, as well as the clinical relevance of and current interest in approaches capable of potentiating or reviving the action of conventional antibiotics [53]. In this respect, empty LPHNPs were employed to probe any intrinsic antimicrobial activity of the delivery platform; CTX-loaded CNPs, CTX-loaded LPHNPs, and co-loaded LPHNPs were also tested to gauge the impact of LPHNP lipid shells on CTX activity, and to ultimately gain insight into the ability of RN7IN6 to potentiate CTX activity when co-administered in the LPHNP platform (Figure 6).

#### 3.6.1. Impact of CNPs and LPHNPs on CTX Activity

When comparing the findings in Figure 6 with those in Table 3, it can be seen that MIC values of CTX in all nanoparticle formulations were higher than those of free CTX either alone or in combination with RN7IN6, as the relevant comparator. As discussed in relation to Figure 5, CTX showed an incomplete release from CNP and LPHNP over a time period aligning with the duration of the antimicrobial efficacy assay, with only 45–51% of CTX being cumulatively released from CNP and LPHNP nanocarriers after 24 h. This could suggest that measured antimicrobial efficacy of CTX in the employed assay is reflective of the free fraction of CTX only, indicating that assays over longer timescales or indeed time-kill kinetic studies should be employed in future work—such longer term setups may fully capture the antibacterial potential of CTX released in a sustained manner, as well as any CTX activity resulting from direct nanoparticle–bacteria interactions (which can take several hours to peak) [54,55]. This suggestion is supported by literature demonstrating a higher MIC of nanoparticle-incorporated antibiotic compared to free drug under conventional MIC assay conditions as a result of exposure of bacteria to differing free/released drug concentrations, and yet an enhanced activity of nanoparticulate drug compared to free over longer timeframes as a result of prolonged drug exposure [56,57]. In the context of combination delivery in particular, it should also be kept in mind that translation of a potent in vitro effect of anti-infective combinations (particularly of actives with differing physicochemical properties) to later pre-clinical or clinical contexts may be hindered if co-administration proves a challenge; this further highlights the utility and necessity of delivery platforms, and the requirement to balance active potency with deliverability.

#### 3.6.2. Impact of LPHNP Lipid Shell on CTX Activity

Comparison of CTX MIC values in CTX-LPHNPs and CTX-CNPs demonstrated that the introduction of a lipid shell led to a 2-fold reduction in antimicrobial activity against Gram-positive *S. aureus* (8 μg/mL cf. 4 μg/mL), but a comparable activity against the Gram-negative *E. coli* (0.25 μg/mL in both cases, Figure 6). An improved relative performance of LPHNPs with a specifically Gram-negative bacteria-relevant lipid shell [58] in *E. coli* is interesting, and may point to a bacteria-dependent impact of the lipid shell on LPHNP anti-infective cargo efficacy—a suggestion that is supported by the notable activity of empty LPHNPs, which showed antimicrobial activity against both bacteria, but a considerably lower MIC in *E. coli* (2 μg/mL) compared to *S. aureus* (64 μg/mL). While the conducted study is preliminary in nature, this observation lends support to the case for deeper investigation of bacteria-relevant lipid shell compositions in LPHNP design for anti-infective applications. As such, a strong focus of current and future work will be on exploring the interaction of nanoparticles employing different bacteria-relevant lipid compositions with various bacterial membrane structures.

#### 3.6.3. Impact of RN7IN6 on CTX Activity in Co-Loaded LPHNPs

The MIC of CTX in co-loaded LPHNPs was observed to be intermediate between that of CTX in LPHNPs and CTX in CNPs in the case of *S. aureus* (Figure 6), suggesting a relatively minor impact of RN7IN6 on the activity of co-delivered CTX. However, similar to the trend identified in Section 3.6.2, co-loaded LPHNPs surprisingly resulted in complete inhibition of *E. coli* growth at all concentrations of CTX/RN7IN6 tested, including the lowest employed concentration combination (0.375 μg/mL CTX, 0.25 μg/mL RN7IN6—Figure 6). This necessitated specification of MIC values for co-loaded CTX and RN7IN6 as being less than the lowest tested concentrations, rather than specific values. This result encouragingly indicates that in the case of *E. coli*, the specific MIC value of CTX incorporated in co-loaded LPHNPs at least approximates, and is likely lower, than the MIC of solely CTX in LPHNPs or in CNPs (MIC 0.25 μg/mL in both cases). Therefore, the current observation may indicate a potentiating effect of RN7IN6 on the antibacterial efficacy of CTX against *E. coli* as a result of co-delivery in LPHNPs, with further investigation required to fully understand the degree of this potentiation. Investigation of the generalizability of this potential activity enhancement to other Gram-negative species is also of considerable future research interest, in light of the specific and considerable challenges associated with the prevention and treatment of resistant Gram-negative bacterial infections [59].

#### 3.6.4. Impact of Co-Loaded LPHNP Delivery on RN7IN6

Comparisons of RN7IN6 activity in co-loaded LPHNPs relative to that in combination with CTX in free form must be made with caution. The wider feasibility study goal to maximize CTX and RN7IN6 loading within LPHNPs demonstrated that the LPHNP platform is able to incorporate a much higher concentration of CTX relative to RN7IN6 (Figure 4), which, coupled with the more potent antibacterial efficacy of free CTX in comparison to free RN7IN6 (Table 3), may lead to a predominantly CTX-mediated impact of this formulation. Further, while not required to address the current aim of probing the CTX-potentiating impact of RN7IN6 in LPHNPs, determination of the antimicrobial efficacy of solely RN7IN6 in LPHNPs would be necessary to accurately gauge the impact of co-delivery on RN7IN6 itself, or indeed, to specifically evaluate synergy. With these caveats in mind, it remains interesting to note that RN7IN6 in co-loaded LPHNPs was observed to have a 1.5-fold (4 μg/mL cf. 6 μg/mL) and at least a 21-fold (<0.25 μg/mL cf. 5.3 μg/mL) lower MIC than free RN7IN6 combined with CTX in *S. aureus* and *E. coli*, respectively (Table 3, Figure 6). This preliminary screen for formulation efficacy can, therefore, be concluded to have fulfilled its purpose, providing strong evidence to support in-depth evaluation of the antimicrobial efficacy of co-loaded LPHNPs in order to establish the extent and scope of their utility.

## 4. Conclusions

In conclusion, this study constitutes a successful proof of concept demonstrating the feasibility of formulating LPHNPs as dual-compartment platforms for co-encapsulation and delivery of anti-infective compounds. Uniformly nanosized LPHNPs optimized for maximal loading of CTX inside polymer cores and RN7IN6 within lipid shells were successfully formulated, using a robust microfluidic mixing method with clear potential for scale-up. The bacteria-relevant LPHNP lipid shell showed a small, but noteworthy impact on CTX release behavior in comparison to CTX-loaded CNP, with RN7IN6 hypothesized to be retained within co-loaded LPHNP lipid shells. Preliminary investigation of the antibacterial efficacy of co-loaded LPHNPs suggested an RN7IN6-mediated enhancement of CTX activity, particularly against the Gram-negative *E. coli*, strongly indicating that further, in-depth investigation of the utility of LPHNPs for anti-infective co-delivery is warranted. Characterization of the interaction of co-loaded LPHNPs with bacterial membrane structures, as well as comprehensive antibacterial efficacy testing aimed at assessing synergistic effects of co-loaded LPHNPs, constitute immediate priorities for further investigation.

## Figures and Tables

**Table 1:** Formulation parameters employed to determine the optimal FRR for empty LPHNP production. Lipid concentration and TFR were fixed based on screening studies detailed in the Supplementary Materials.

Formulation	Lipid Concentration (mg/mL)	Weight Ratio (Lipid:Chitosan)	TFR (mL/min)	FRR (CNP:Lipid)
E2:1	0.5	0.43:1	20	2:1
E3:1	0.5	0.32:1	20	3:1
E4:1	0.5	0.26:1	20	4:1
E5:1	0.5	0.21:1	20	5:1
E6:1	0.5	0.18:1	20	6:1

**Table 2:** Microfluidic process parameters employed to manufacture CTX and RN7IN6 co-loaded LPHNPs with varying loading concentrations of RN7IN6.

		Organic Phase			
Formulation	Aqueous Phase	RN7IN6 (mg/mL)	Lipid (mg/mL)	TFR (mL/min)	FRR (CTX-Loaded CNPs:RN7IN6 in Lipid
0.5 R	CTX-CNP	0.5	0.5	20	5:1
0.75 R		0.75			
1 R		1			
1.5 R		1.5			
2 R		2			

**Table 3:** Minimum inhibitory concentration (MIC) values for RN7IN6 and CTX when tested separately, and in combination, against *S. aureus* and *E. coli*, and calculation of fractional inhibitory concentration indices (FICI) from these values. In both cases, partial synergism was indicated. Results are given as mean ± SD of testing in duplicate and triplicate for *S. aureus* and *E. coli*, respectively.

	MIC-Separate (μg/mL)		MIC-in Combination (μg/mL)			
Bacteria	RN7IN6	CTX	RN7IN6	CTX	FICI	Effect
*S. aureus*	16.00 ± 0.00	2.00 ± 0.00	6.00 ± 2.83	0.750 ± 0.353	0.75 ± 0.00	Partial synergism
*E. coli*	32.00 ± 0.00	0.10 ± 0.04	5.33 ± 2.31	0.052 ± 0.018	0.67 ± 0.07	Partial synergism

## Data Availability

The original contributions presented in this study are included in the article/Supplementary Material. Further inquiries can be directed to the corresponding author.

## Supplementary Materials

The following supporting information can be downloaded at: https://www.mdpi.com/article/10.3390/pharmaceutics18010013/s1, Supplementary Methods; Table S1: Set of 18 CNP formulations resulting from Taguchi design of experiments L18 orthogonal array; Table S2: Set of 9 formulations employed to investigate the effect of initial lipid concentration, TFR and FRR on empty LPHNP size, PDI, and zeta potential; Table S3: Set of 30 formulations employed to further investigate the effect of initial lipid concentration, TFR and FRR on empty LPHNP size, PDI, and zeta potential; Supplementary Results; Table S4: Empty CNP size, PDI, and zeta potential of 18 formulations prepared according to Taguchi L18 orthogonal array design of experiments; Table S5; Optimal process parameters for empty CNPs; Figure S1: Size and PDI of empty CNP (Formulation G) manufacture to further assess FRR impact; Figure S2: The effect of increasing CTX loading concentrations on size and PDI and zeta potential of CNP; Figure S3: The effect of increasing CTX loading concentrations on encapsulation efficiency and incorporated drug concentration for CNP; Figure S4: Size and PDI and zeta potential of 9 empty LPHNP formulations; Figure S5: Size, PDI, and zeta potential of 30 empty LPHNP formulations; Figure S6: One-way ANOVA analysis for size, PDI, and zeta potential with Tukey’s post hoc comparison performed on CTX and RN7IN6 co-loaded LPHNPs and comparator formulations; Figure S7: One-way analysis for encapsulation efficiency and incorporated active concentration with Tukey’s post hoc comparison performed on CTX and RN7IN6 co-loaded LPHNPs and comparator formulations.

## Abbreviations

The following abbreviations are used in this manuscript:

AMP	Antimicrobial peptide
AMR	Antimicrobial resistance
CL	1′,3′-bis [1,2-dioleoyl-sn-glycero-3-phospho]- glycerol, tetraoleoyl cardiolipin
CNP	Chitosan nanoparticle
CTX	Cefotaxime
DMF	N,N-dimethylformamide
EE%	Encapsulation efficiency
FIC	Fractional inhibitory concentration
FICI	Fractional inhibitory concentration index
FRR	Flow rate ratio
LPHNP	Lipid-polymer hybrid nanoparticle
MHB2	Mueller Hinton broth 2
MIC	Minimum inhibitory concentration
PBS	Phosphate-buffered saline
PDI	Polydispersity index
POPE	1-palmitoyl-2-oleoyl-sn-glycero-3-phosphoethanolamine
POPG	1-palmitoyl-2-oleoyl-sn-glycero-3-phospho-(1′-rac-glycerol) (sodium salt)
TFA	Trifluoroacetic acid
TFR	Total flow rate

## References

[1] O’Neill J. (2016). Review on Antimicrobial Resistance. Tackling Drug Resistant Infections Globally: Final Report and Recommendations.

[2] Murray C.J.L., Ikuta K.S., Sharara F., Swetschinski L., Robles Aguilar G., Gray A., Han C., Bisignano C., Rao P., Wool E. (2022). Global burden of bacterial antimicrobial resistance in 2019: A systematic analysis. Lancet.

[3] Maher C., Hassan K.A. (2023). The Gram-negative permeability barrier: Tipping the balance of the in and the out. mBio.

[4] Gargate N., Laws M., Rahman K.M. (2025). Current economic and regulatory challenges in developing antibiotics for Gram-negative bacteria. npj Antimicrob. Resist..

[5] World Health Organisation (2024). 2023 Antibacterial Agents in Clinical and Preclinical Development: An Overview and Analysis.

[6] Gajbhiye K.R., Salve R., Narwade M., Sheikh A., Kesharwani P., Gajbhiye V. (2023). Lipid polymer hybrid nanoparticles: A custom-tailored next-generation approach for cancer therapeutics. Mol. Cancer.

[7] Mohanty A., Uthaman S., Park I.-K. (2020). Utilization of polymer-lipid hybrid nanoparticles for targeted anti-cancer therapy. Molecules.

[8] Shen S., Li T., Fan J., Shao Q., Dong H., Xu X., Mo R. (2023). Lipid-polymer hybrid nanoparticle with cell-distinct drug release for treatment of stemness-derived resistant tumor. Acta Pharm. Sin. B.

[9] Baek J.-S., Tan C.H., Ng N.K.J., Yeo Y.P., Rice S.A., Loo S.C.J. (2018). A programmable lipid-polymer hybrid nanoparticle system for localized, sustained antibiotic delivery to Gram-positive and Gram-negative bacterial biofilms. Nanoscale Horiz..

[10] Fernández-Borbolla A., García-Hevia L., Fanarraga M.L. (2024). Cell membrane-coated nanoparticles for precision medicine: A comprehensive review of coating techniques for tissue-specific therapeutics. Int. J. Mol. Sci..

[11] Gao F., Xu L., Yang B., Fan F., Yang L. (2019). Kill the real with the fake: Eliminate intracellular *Staphylococcus aureus* using nanoparticle coated with its extracellular vesicle membrane as active-targeting drug carrier. ACS Infect. Dis..

[12] Tomlinson S., Taylor P.W., Luzio J.P. (1989). Transfer of phospholipid and protein into the envelope of Gram-negative bacteria by liposome fusion. Biochemistry.

[13] Wu S., Huang Y., Yan J., Li Y., Wang J., Yang Y.Y., Yuan P., Ding X. (2021). Bacterial outer membrane-coated mesoporous silica nanoparticles for targeted delivery of antibiotic rifampicin against Gram-negative bacterial infection in vivo. Adv. Funct. Mater..

[14] Khan M., Madni A., Tahir N., Parveen F., Khan S., Jan N., Ali A., Abdurrahim M., Farooq U., Khan M. (2020). Co-delivery of curcumin and cisplatin to enhance cytotoxicity of cisplatin using lipid-chitosan hybrid nanoparticles. Int. J. Nanomed..

[15] Murugaiyan J., Kumar P.A., Rao G.S., Iskandar K., Hawser S., Hays J.P., Mohsen Y., Adukkadukkam S., Awuah W.A., Jose R.A.M. (2022). Progress in alternative strategies to combat antimicrobial resistance: Focus on antibiotics. Antibiotics.

[16] Rajamanickam K., Yang J., Sakharkar M.K. (2019). A novel antimicrobial–phytochemical conjugate with antimicrobial activity against *Streptococcus uberis*, *Enterococcus faecium*, and *Enterococcus faecalis*. Front. Pharmacol..

[17] Jindal H.M., Le C.F., Mohd Yusof M.Y., Velayuthan R.D., Lee V.S., Zain S.M., Isa D.M., Sekaran S.D. (2015). Antimicrobial activity of novel synthetic peptides serived from indolicidin and ranalexin against *Streptococcus pneumoniae*. PLoS ONE.

[18] D’Aloisio V., Schofield A., Kendall D.A., Hutcheon G.A., Coxon C.R. (2024). The development and optimisation of an HPLC-based in vitro serum stability assay for a calcitonin gene-related peptide receptor antagonist peptide. J. Pept. Sci..

[19] Graef F., Gordon S., Lehr C.-M. (2016). Anti-infectives in drug delivery—Overcoming the Gram-negative bacterial cell envelope. Curr. Top. Microbiol. Immunol..

[20] Roces C.B., Lou G., Jain N., Abraham S., Thomas A., Halbert G.W., Perrie Y. (2020). Manufacturing considerations for the development of lipid nanoparticles using microfluidics. Pharmaceutics.

[21] Needs S.H., Saiprom N., Rafaque Z., Imtiaz W., Chantratita N., Runcharoen C., Thammachote J., Anun S., Peacock S.J., Ray P. (2022). Miniaturised broth microdilution for simplified antibiotic susceptibility testing of Gram negative clinical isolates using microcapillary devices. Analyst.

[22] Bellio P., Fagnani L., Nazzicone L., Celenza G. (2021). New and simplified method for drug combination studies by checkerboard assay. MethodsX.

[23] Fatsis-Kavalopoulos N., Sánchez-Hevia D.L., Andersson D.I. (2024). Beyond the FIC index: The extended information from fractional inhibitory concentrations (FICs). J. Antimicrob. Chemother..

[24] Gui K., Zhang X., Chen F., Ge Z., Zhang S., Qi X., Sun J., Yu Z. (2019). Lipid-polymer nanoparticles with CD133 aptamers for targeted delivery of all-trans retinoic acid to osteosarcoma initiating cells. Biomed. Pharmacother..

[25] Li P., Chen X., Shen Y., Li H., Zou Y., Yuan G., Hu P., Hu H. (2019). Mucus penetration enhanced lipid polymer nanoparticles improve the eradication rate of *Helicobacter pylori* biofilm. J. Control. Release.

[26] Lin J., Cai Q., Tang Y., Xu Y., Wang Q., Li T., Xu H., Wang S., Fan K., Liu Z. (2018). PEGylated lipid bilayer coated mesoporous silica nanoparticles for co-delivery of paclitaxel and curcumin: Design, characterization and its cytotoxic effect. Int. J. Pharm..

[27] Liu D., Lian Y., Fang Q., Liu L., Zhang J., Li J. (2018). Hyaluronic-acid-modified lipid-polymer hybrid nanoparticles as an efficient ocular delivery platform for moxifloxacin hydrochloride. Int. J. Biol. Macromol..

[28] Hu Y., Hoerle R., Ehrich M., Zhang C. (2015). Engineering the lipid layer of lipid–PLGA hybrid nanoparticles for enhanced in vitro cellular uptake and improved stability. Acta Biomater..

[29] Wan F., Nylander T., Klodzinska S.N., Foged C., Yang M., Baldursdottir S.G., Nielsen H.M. (2018). Lipid shell-enveloped polymeric nanoparticles with high integrity of lipid shells improve mucus penetration and interaction with cystic fibrosis-related bacterial biofilms. ACS Appl. Mater. Interfaces.

[30] Jiang L., Lee H.W., Loo S.C.J. (2020). Therapeutic lipid-coated hybrid nanoparticles against bacterial infections. RSC Adv..

[31] Tormena N., Pilizota T., Voïtchovsky K. (2025). A minimalist model lipid system mimicking the biophysical properties of *Escherichia coli*’s inner membrane. Langmuir.

[32] Drost M., Diamanti E., Fuhrmann K., Goes A., Shams A., Haupenthal J., Koch M., Hirsch A.K.H., Fuhrmann G. (2022). Bacteriomimetic liposomes improve antibiotic activity of a novel energy-coupling factor transporter inhibitor. Pharmaceutics.

[33] Paez-Perez M., Russell I.A., Cicuta P., Di Michele L. (2022). Modulating membrane fusion through the design of fusogenic DNA circuits and bilayer composition. Soft Matter.

[34] Zhang L., Chan J.M., Gu F.X., Rhee J.-W., Wang A.Z., Radovic-Moreno A.F., Alexis F., Langer R., Farokhzad O.C. (2008). Self-assembled lipid–polymer hybrid nanoparticles: A robust drug delivery platform. ACS Nano.

[35] Anwer M.K., Ali E.A., Iqbal M., Ahmed M.M., Aldawsari M.F., Saqr A.A., Ansari M.N., Aboudzadeh M.A. (2022). Development of sustained release baricitinib loaded lipid-polymer hybrid nanoparticles with improved oral bioavailability. Molecules.

[36] Javaid S., Ahmad N.M., Mahmood A., Nasir H., Iqbal M., Ahmad N., Irshad S. (2021). Cefotaxime loaded polycaprolactone based polymeric nanoparticles with antifouling properties for in-vitro drug release applications. Polymers.

[37] Shafique M., Ur Rehman M., Kamal Z., Alzhrani R.M., Alshehri S., Alamri A.H., Bakkari M.A., Sabei F.Y., Safhi A.Y., Mohammed A.M. (2023). Formulation development of lipid polymer hybrid nanoparticles of doxorubicin and its in-vitro, in-vivo and computational evaluation. Front. Pharmacol..

[38] Jindal H.M., Zandi K., Ong K.C., Velayuthan R.D., Rasid S.M., Samudi Raju C., Sekaran S.D. (2017). Mechanisms of action and in vivo antibacterial efficacy assessment of five novel hybrid peptides derived from indolicidin and ranalexin against *Streptococcus pneumoniae*. PeerJ.

[39] Lee A.L.Z., Wang Y., Ye W.-H., Yoon H.S., Chan S.Y., Yang Y.-Y. (2008). Efficient intracellular delivery of functional proteins using cationic polymer core/shell nanoparticles. Biomaterials.

[40] Parashar S., Chauhan C., Rajasekharan A., Rautela J., Jain T., Raza K. (2022). An augmented method for collecting PLGA nanoparticles and the fabrication of 1,3,4,6-tetra-O-acetyl-2-azido-2-deoxy-D-glucopyranose (Ac42AzGlc)-loaded PLGA nanoparticles for efficient and prospective in vivo metabolic processing. Front. Bioeng. Biotechnol..

[41] Filippov S.K., Khusnutdinov R., Murmiliuk A., Inam W., Zakharova L.Y., Zhang H., Khutoryanskiy V.V. (2023). Dynamic light scattering and transmission electron microscopy in drug delivery: A roadmap for correct characterization of nanoparticles and interpretation of results. Mater. Horiz..

[42] Maguire C.M., Rösslein M., Wick P., Prina-Mello A. (2018). Characterisation of particles in solution—A perspective on light scattering and comparative technologies. Sci. Technol. Adv. Mater..

[43] Torcato I.M., Huang Y.-H., Franquelim H.G., Gaspar D., Craik D.J., Castanho M.A.R.B., Troeira Henriques S. (2013). Design and characterization of novel antimicrobial peptides, R-BP100 and RW-BP100, with activity against Gram-negative and Gram-positive bacteria. Biochim. Biophys. Acta Biomembr..

[44] Hsu J.C.Y., Yip C.M. (2007). Molecular dynamics simulations of indolicidin association with model lipid bilayers. Biophys. J..

[45] Nielsen J.E., Bjørnestad V.A., Lund R. (2018). Resolving the structural interactions between antimicrobial peptides and lipid membranes using small-angle scattering methods: The case of indolicidin. Biophys. J..

[46] Lee H.W., Kharel S., Loo S.C.J. (2022). Lipid-coated hybrid nanoparticles for enhanced bacterial biofilm penetration and antibiofilm efficacy. ACS Omega.

[47] Wang H., Zhao P., Su W., Wang S., Liao Z., Niu R., Chang J. (2010). PLGA/polymeric liposome for targeted drug and gene co-delivery. Biomaterials.

[48] Murzyn K., Róg T., Pasenkiewicz-Gierula M. (2005). Phosphatidylethanolamine-phosphatidylglycerol bilayer as a model of the inner bacterial membrane. Biophys. J..

[49] Unsay J.D., Cosentino K., Subburaj Y., García-Sáez A.J. (2013). Cardiolipin effects on membrane structure and dynamics. Langmuir.

[50] World Health Organisation (2024). WHO Bacterial Priority Pathogens List, 2024: Bacterial Pathogens of Public Health Importance to Guide Research, Development and Strategies to Prevent and Control Antimicrobial Resistance.

[51] Roque-Borda C.A., Zhang Q., Nguyen T.P.T., Nguyen T.T.H., Medhi H., de Souza Rodrigues H.L., Canales Carnero C.S., Sutherland D., Helmy N.M., Araveti P.B. (2025). Synergistic combinations of antimicrobial peptides and conventional antibiotics: A strategy to delay resistance emergence in WHO priority bacteria. Pharmacol. Rev..

[52] Tong Z., Zhang Y., Ling J., Ma J., Huang L., Zhang L. (2014). An in vitro study on the effects of nisin on the antibacterial activities of 18 antibiotics against *Enterococcus faecalis*. PLoS ONE.

[53] Tabcheh J., Vergalli J., Davin-Régli A., Ghanem N., Pages J.-M., Al-Bayssari C., Brunel J.M. (2023). Rejuvenating the activity of usual antibiotics on resistant Gram-negative bacteria: Recent issues and perspectives. Int. J. Mol. Sci..

[54] Mugabe C., Halwani M., Azghani A.O., Lafrenie R.M., Omri A. (2006). Mechanism of enhanced activity of liposome-entrapped aminoglycosides against resistant strains of *Pseudomonas aeruginosa*. Antimicrob. Agents Chemother..

[55] Sachetelli S., Khalil H., Chen T., Beaulac C., Sénéchal S., Lagacé J. (2000). Demonstration of a fusion mechanism between a fluid bactericidal liposomal formulation and bacterial cells. Biochim. Biophys. Acta.

[56] Cheow W.S., Chang M.W., Hadinoto K. (2010). Antibacterial efficacy of inhalable levofloxacin-loaded polymeric nanoparticles against *E. coli* biofilm cells: The effect of antibiotic release profile. Pharm. Res..

[57] Jiang L., Greene M.K., Insua J.L., Pessoa J.S., Small D.M., Smyth P., McCann A.P., Cogo F., Bengoechea J.A., Taggart C.C. (2018). Clearance of intracellular *Klebsiella pneumoniae* infection using gentamicin-loaded nanoparticles. J. Control. Release.

[58] Graef F., Vukosavljevic B., Michel J.-P., Wirth M., Ries O., De Rossi C., Windbergs M., Rosilio V., Ducho C., Gordon S. (2016). The bacterial cell envelope as delimiter of anti-infective bioavailability—An in vitro permeation model of the Gram-negative bacterial inner membrane. J. Control. Release.

[59] Macesic N., Uhlemann A.-C., Peleg A.Y. (2025). Multidrug-resistant Gram-negative bacterial infections. Lancet.

