# Supplementary material for: Dual Core-Shell Loaded Lipid-Polymer Hybrid Nanoparticles as Combination Anti-Infective Delivery Platforms
Source: Pharmaceutics. 2025 Dec 22;18(1):13. doi: 10.3390/pharmaceutics18010013 (PMC12845076; doi:10.3390/pharmaceutics18010013)
Supplement: Supplementary file 1 [file pharmaceutics-18-00013-s001.zip › pharmaceutics-4014105-supplementary.pdf]

Supplementary Materials

Dual Core-Shell Loaded Lipid-Polymer Hybrid Nanoparticles as Combination Anti-infective Delivery Platforms

Valeria Carini [1], Giulia Scagnetti [1], Joanne Foulkes [1], Katie Evans [1], Imran Saleem [1] and Sarah Gordon [2,*]

1. Supplementary Methods

1.1 Empty Chitosan Nanoparticle (CNP) Preparation

Chitosan hydrochloride (MW 30-400 kDa, degree of deacetylation 80-95%, Heppe Medical Chitosan GmbH, Halle, Germany) was dissolved in purified water at concentrations of 0.25-1 mg/mL and left to stir overnight at room temperature. Solutions were then adjusted to pH 5 using NaOH, in accordance with previous studies performed by the authors, and filtered through Whatman no. 40 filter paper (8 μm, Sigma-Aldrich, MO, USA). Solutions of sodium tripolyphosphate (TPP, Sigma-Aldrich, MO, USA), used as a crosslinker, were prepared by dissolution in purified water to produce concentrations of 0.25-1 mg/mL.

CNP formation was achieved using the Nanoassemblr® Benchtop instrument equipped with a staggered herringbone mixer cartridge (Precision Nanosystems Inc., Vancouver, Canada). Briefly, chitosan and TPP solutions were loaded into disposable and compatible syringes and injected into separate inlets of the cartridge following specification of suitable process parameters – namely, total flow rate (TFR) and flow rate ratio (FRR) (see Table S1). CNP formed spontaneously as a result of controlled mixing were collected from the outlet port of the cartridge in 15 mL Falcon tubes. Size (Z-average), polydispersity index (PDI) and zeta potential of CNP was measured as described in section 2.5 of the main manuscript.

1.1.1 Optimization of Empty CNP – Taguchi Design of Experiments

To allow for evaluation of the influence of formulation parameters on CNP size and PDI while minimizing the number of experiments to be conducted, a Taguchi design of experiments approach was employed using Minitab® version 20 statistical software (Minitab, LLC). A Taguchi L18 orthogonal array design was constructed composed of 4 variables (chitosan concentration, TPP concentration, TFR and FRR) set at 3 or 6 levels, as indicated in Table S1. The software output of 18 formulations was then prepared in triplicate.

The optimum conditions were set as a high signal to noise (S/N) ratio, corresponding to a minimum variance of the outcome and a better performance, with the target output parameters being particle size and PDI. The optimization of the particle size and PDI was performed using the Taguchi 'smaller-is-better' criterion to achieve a particle size and a PDI as small as possible.

Table S1. Set of 18 CNP formulations resulting from Taguchi design of experiments L18 orthogonal array.

Formulation	Chitosan (mg/mL)	TPP (mg/mL)	TFR (mL/min)	FRR (Chitosan:TPP)
A	0.25	0.25	0.64	2:1
B	0.50	0.50	0.80	2:1
C	1.00	1.00	1.00	2:1
D	0.25	0.50	0.64	3:1
E	0.50	1.00	0.80	3:1
F	1.00	0.25	1.00	3:1
G	0.50	0.25	0.64	4:1
H	1.00	0.50	0.80	4:1
I	0.25	1.00	1.00	4:1
J	1.00	1.00	0.64	5:1
K	0.25	0.25	0.80	5:1
L	0.50	0.50	1.00	5:1
M	0.50	1.00	0.64	6:1
N	1.00	0.25	0.80	6:1
O	0.25	0.50	1.00	6:1
P	1.00	0.50	0.64	7:1
Q	0.25	1.00	0.80	7:1
R	0.50	0.25	1.00	7:1

1.1.2 Empty CNP – Further FRR Investigation

Further to the formulations described in Table S1, a further investigation of FRR was carried out to obtain a small CNP size and PDI and increase the S/N ratio. The best formulation determined following conduction of the design of experiments (Table S1), was chosen as a starting point with all parameters of this formulation fixed apart from FRR, which was varied from 1:1 to 7:1.

1.2 Cefotaxime-loaded CNPs – Preparation, Optimization and Characterization

Cefotaxime (CTX)-loaded CNPs were manufactured by microfluidic mixing, employing optimal FRR, TFR, chitosan and TPP concentrations identified for empty CNPs as baseline parameters. The impact of varying CTX loading concentration on CNP size, PDI, zeta potential and encapsulation efficiency (EE%) was then investigated, by adding CTX to either chitosan or TPP solutions to give drug concentrations of 0.3-3 mg/mL. Solutions were then injected into separate inlets of the microfluidic cartridge and CTX-loaded CNP recovered in a 15 mL Falcon tube. CTX-loaded CNP were characterized for size, PDI and zeta potential as described in the main manuscript (section 2.5). CTX EE% was calculated following centrifugal ultrafiltration, HPLC analysis of unentrapped CTX and employment of an indirect calculation method to calculate EE% as described in the main manuscript (sections 2.6 and 2.7) with the following variations: column oven temperature 30 °C; injection volume 5 μL; mobile phase 40:60 mix of A:B, with A = 0.2% formic acid in water and B = acetonitrile, at a flow rate of 1 mL/min (total run time 3.30 min); UV detection wavelength 308 nm..

1.3 Empty Lipid-Polymer Hybrid Nanoparticles - Preparation, Optimization and Characterization

Empty Lipid-Polymer Hybrid Nanoparticles (LPHNPs) were prepared by microfluidic mixing, as described in detail in the main manuscript, utilizing unloaded CNPs (optimized as described above) as core structures and a bacteria-relevant lipid mixture as shell components. An initial screening of LPHNP manufacturing parameters was first performed to assess the influence of the initial lipid concentration, the TFR and the FRR between CNPs in dispersion and the lipid solution on LPHNP characteristics. The initial screening was performed using a Taguchi L9 orthogonal array design with 3 variables set at 3 levels (Table S2). Produced empty LPHNP were characterized for size, PDI and zeta potential as described in section 2.5 of the main manuscript.

Table S2. Set of 9 formulations employed to investigate the effect of initial lipid concentration, TFR and FRR on empty LPHNP size, PDI and zeta potential. Formulations are named as Tag1-Tag9 to reflect the Taguchi design of experiments employed.

Formulation	Lipid concentration (mg/mL)	TFR (mL/min)	FRR (CNP:lipid)
Tag1	2	5	2:1
Tag2	2	10	3:1
Tag3	2	20	4:1
Tag4	4	5	3:1
Tag5	4	10	4:1
Tag6	4	20	2:1
Tag7	8	5	4:1
Tag8	8	10	2:1
Tag9	8	20	3:1

Following this initial screening, a full factorial design investigating one-variable-at-a-time impact on empty LPHNP physicochemical characteristics was conducted (Table S3). This further screen employed 3 additional, lower lipid concentrations in comparison to the initial study (2, 1 and 0.5 mg/mL) as well as 5 different FRR (2:1, 3:1, 4:1, 5:1 and 6:1) and 2 levels of TFR (10 and 20 mL/min). The size, PDI and zeta potential of produced empty LPHNP was determined as described in the main manuscript.

Table S3. Set of 30 formulations employed to further investigate the effect of initial lipid concentration, TFR and FRR on empty LPHNP size, PDI and zeta potential.

Formulation	Lipid concentration (mg/mL)	TFR (mL/min)	FRR (CNP:lipid)
A.1	2	10, 20	2:1
A.2	2	10, 20	3:1
A.3	2	10, 20	4:1
A.4	2	10, 20	5:1
A.5	2	10, 20	6:1
B.1	1	10, 20	2:1
B.2	1	10, 20	3:1
B.3	1	10, 20	4:1
B.4	1	10, 20	5:1
B.5	1	10, 20	6:1
C.1	0.5	10, 20	2:1
C.2	0.5	10, 20	3:1
C.3	0.5	10, 20	4:1
C.4	0.5	10, 20	5:1
C.5	0.5	10, 20	6:1

1.4 RN7IN6 Synthesis

The Peptide Companion Excel spreadsheet was used to predict difficult couplings within the peptide sequence of the antimicrobial peptide RN7IN6 (http://www.spyderinstitute.com/software.html). RN7IN6 was examined with this tool to predict challenging coupling steps requiring double coupling to increase the likelihood of high synthesis yields.

RN7IN6 was prepared using an automated 9-fluorenylmethyloxycarbonyl solid-phase peptide synthesis (Fmoc-SPPS) method on a Liberty Blue microwave-assisted Peptide Synthesizer (CEM, USA) using a method adapted from D′Aloisio *et al.* (reference details in main manuscript). Solid phase synthesis was conducted on a 0.1 mmol scale using Rink Amide ProTide resin (179 mg, 0.56 mmol/g loading) employing the required Fmoc L-amino acids (0.2 M in dimethylformamide (DMF; 5eq.), and N,N′-Diisopropylcarbodiimide (DIC, 1 M in DMF; 10 eq.), Oxyma Pure (1 M in DMF; 5 eq.) and piperidine (20% *v/v* in DMF; 4 mL) as activator, racemization suppressor and deprotection reagent, respectively. The peptide synthesis was carried out using a standard coupling procedure employing double coupling of each amino acid (2.5 min, 90 ^{0}C) and Fmoc deprotection (2 min, 90 ^{0}C). Fmoc-Arg(Pbf)-OH was coupled with triple coupling under mild conditions (75 ^{0}C), while the subsequent Fmoc-Trp(Boc)- OH was coupled with triple coupling (2.5 min, 90 ^{0}C). Upon completion of SPPS synthesis, the resin was washed with DIC and then shrunk with diethyl ether. Subsequently, the peptide was cleaved from the resin using 4 mL of cleavage solution (trifluoroacetic acid (TFA), triisopropylsilane (TIPS), water (8:1:1 v/v)) under regular shaking (Eppendorf Thermomixer comfort) at room temperature for 3 h. The crude peptide was precipitated dropwise in a cold mixture of diethyl and petroleum ether (1:1 v/v) and the obtained suspension was centrifuged (10 min, 3500 rpm, Eppendorf centrifuge 5804r, Germany). The pellet was then washed twice using a cold mixture of diethyl and petroleum ether (1:1 v/v) to remove the residual TFA. Finally, the crude peptide was dissolved in water, flash-frozen using liquid nitrogen, and lyophilized.

1.5 RN7IN6 Purification

Produced, crude RN7IN6 was purified using preparative HPLC. Crude samples (10 mg/mL in H_2O/acetonitrile (ACN) 80/20) were purified using an Agilent Infinity 1260 equipped with a Waters XBridge Peptide BEH C18 Prep 130 Å column (5 µm particle size, 10 x 150 mm). A gradient elution method was employed using H_2O/ACN (30 min: from 45 to 60% ACN, with 0.1% TFA, followed by a 100% ACN for 5 minutes) at a flow rate of 8 mL/min and 50-600 µL injection volumes. The column was kept at room temperature and signals were recorded at 215 nm. Isolated pure compound solutions were concentrated by evaporating residual organic solvents under an N_2 line. The resulting aqueous solutions were analyzed for purity by HPLC (see section 1.6 below), flash frozen with liquid nitrogen, freeze-dried, and stored at -20 °C.

1.6 RN7IN6 Analysis of Purity

Analytical purity of RN7IN6 was determined by HPLC as described in section 2.7 of the main manuscript. The purity percentage of the RN7IN6 was calculated by determining the peak area of the main product relative to the sum of all (main + side) product areas. RN7IN6 purity of >95% was then employed for studies described in the main manuscript.

1.7 Statistical Analysis

All statistical analyses were performed using IBM SPSS statistical software (Version 30.0.0.0, IBM Corp., NY, USA). One-way ANOVA with Tukey's post hoc test was employed to compare formulations obtained as part of the further investigation of FRR impact on empty CNP characteristics, as well as for analysis of CTX-loaded CNP characteristics. A p value of <0.05 was taken as indicating statistical significance throughout the studies.

2. Supplementary Results

2.1 Empty Chitosan Nanoparticle (CNP) Preparation, Optimization and Characterization

Table S4. Empty CNP size, PDI and zeta potential of 18 formulations prepared according to Taguchi L18 orthogonal array design of experiments. Results represent mean ± SD, n =3. Formulation G (bold) was identified as the optimal formulation based on experimental data.

Formulation	Size (nm)	PDI	Zeta Potential (mV)
A	4023.67 ± 2182.36	0.35 ±0.16	4.41 ± 0.81
B	4290.67 ± 1295.92	0.42 ± 0.28	6.63 ± 2.65
C	3006.00 ± 466.66	0.62 ± 0.23	1.07 ± 1.28
D	3818.00 ± 532.25	0.45 ± 0.44	7.63 ± 3.04
E	3752.67 ± 1260.42	0.52 ± 0.16	0.93 ± 0.47
F	130.47 ± 14.84	0.36 ± 0.07	35.75 ± 0.78
**G**	**105.96 ± 11.00**	**0.29 ± 0.04**	**31.30 ± 1.70**
H	158.60 ± 7.83	0.36 ± 0.05	33.20 ± 3.54
I	4022.33 ± 715.06	0.48 ± 0.08	3.28 ± 2.64
J	215.10 ± 82.31	0.48 ± 0.15	32.45 ± 0.35
K	102.50 ± 39.17	0.39 ± 0.10	13.00 ± 2.55
L	109.52 ± 18.26	0.35 ± 0.06	25.95 ± 2.90
M	220.47 ± 37.51	0.47 ± 0.04	24.95 ± 2.19
N	148.71 ± 29.15	0.39 ± 0.06	33.90 ± 4.24
O	89.15 ± 26.29	0.38 ± 0.05	21.40 ± 1.13
P	198.83 ± 58.01	0.38 ± 0.04	35.60 ± 1.27
Q	2360.33 ± 417.90	0.36 ± 0.16	8.47 ± 2.31
R	126.39 ± 59.55	0.33 ± 0.03	10.14 ± 8.44

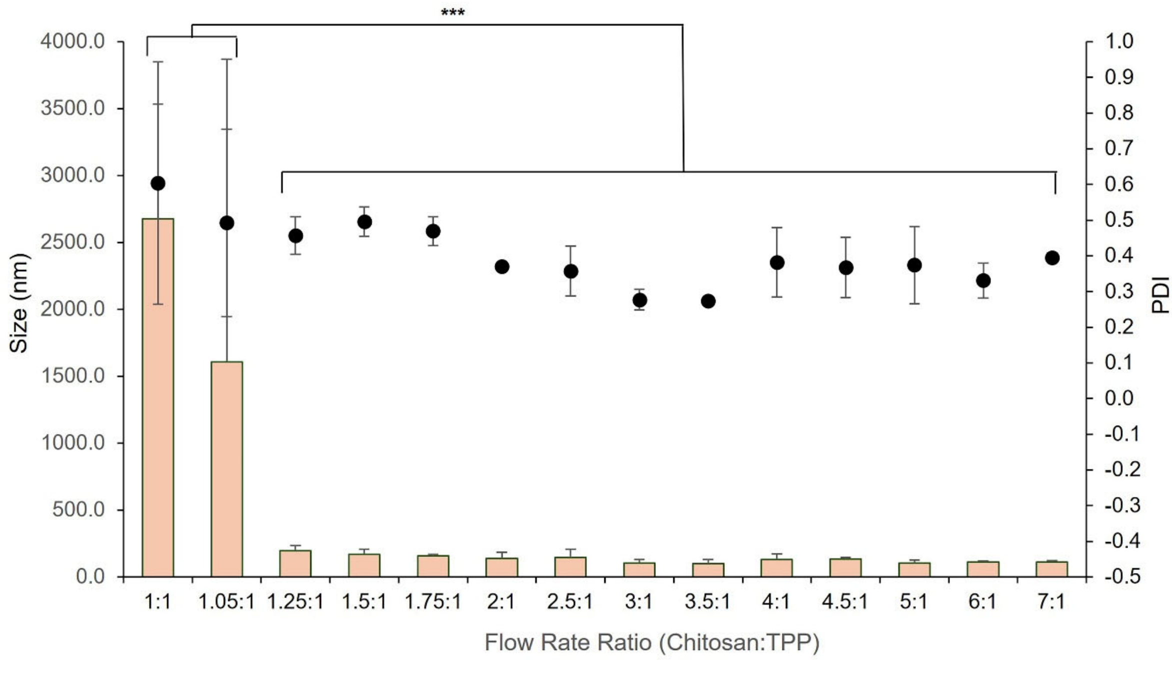

Figure S1. Size and PDI of empty CNP (Formulation G) manufactured to further assess FRR impact. From this data, a FRR of 3.5:1 was identified as being optimal. Results represent mean ± SD, n = 3. Statistical significance relates to CNP size (PDI results did not show any significant differences), *** = $p<0.001$.

Findings presented in Table S4 demonstrated that Formulation G (parameters for preparation listed in Table S1) yielded the smallest and most uniform particle size; further investigation of the impact of fine-tuning FRR on the size and PDI of this formulation (Figure S1) led to selection of 3.5:1 as an optimal FRR. Finalized, optimal process parameters for preparation of empty CNPs to be utilized as polymer cores of empty LPHNPs in the main manuscript, and further employed to manufacture CTX-loaded CNPs, are summarized in Table S5.

Table S5. Optimal process parameters for empty CNPs, further employed to manufacture CTX-loaded CNPs.

Parameter	Value
Chitosan (mg/mL)	0.50
TPP (mg/mL)	0.25
FRR (Chitosan:TPP)	3.5:1
TFR (mL/min)	0.64

2.2 CTX-loaded CNP Preparation, Optimization and Characterization

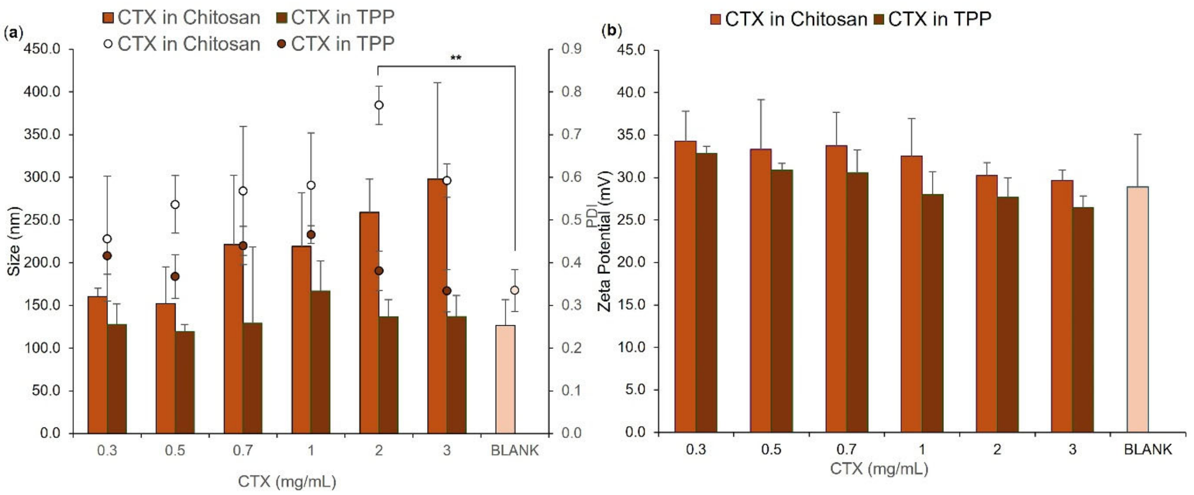

Figure S2. The effect of increasing CTX loading concentrations on size and PDI (**a**) and zeta potential (**b**) of CNP produced by microfluidic mixing using parameters specified in Table S5, and incorporating CTX in either chitosan or TPP solution, with empty CNP shown for comparison ('BLANK'). Results represent mean ± SD, n = 3. ** = p<0.01.

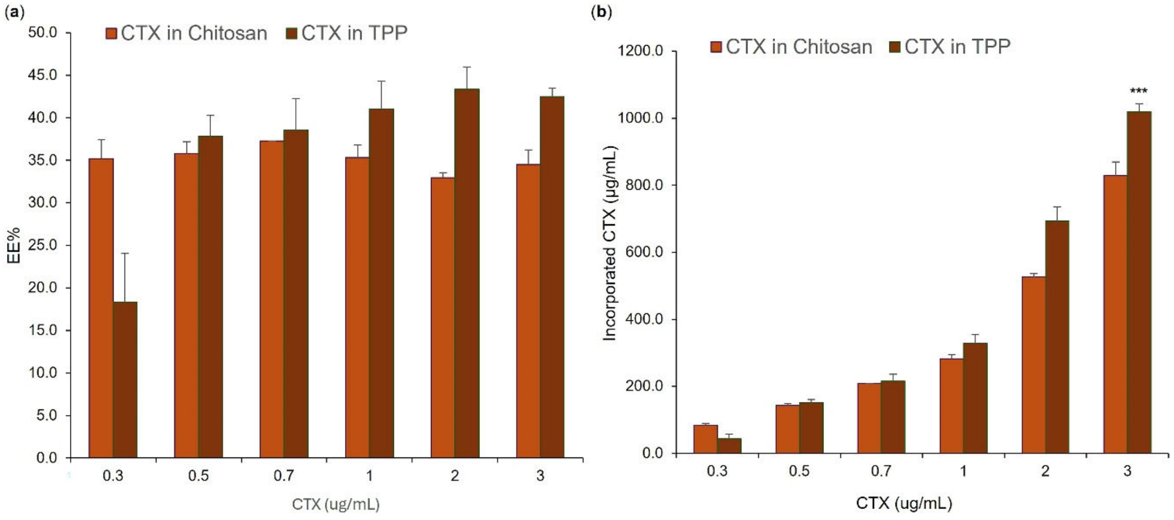

Figure S3. The effect of increasing CTX loading concentrations on encapsulation efficiency (EE%, **a**) and incorporated drug concentration (**b**) for CNP produced by microfluidic mixing using parameters specified in Table S5, and incorporating CTX in either chitosan or TPP solution. Results represent mean ± SD, n = 3. *** = p<0.001 for CNP prepared by loading 3 mg/mL CTX into TPP solution, in comparison to all other formulations.

From findings presented in Figure S2 and S3, loading conditions of 3 mg/mL CTX dissolved in TPP were selected as optimal for preparation of CTX-loaded CNP (utilized as CTX-loaded polymer cores of LPHNP in the main manuscript). This loading condition was shown to give the highest drug loading (Figure S3b), with no significant difference in size, PDI or zeta potential to empty CNP (Figure S2).

2.3 Empty LPHNP Preparation, Optimization and Characterization

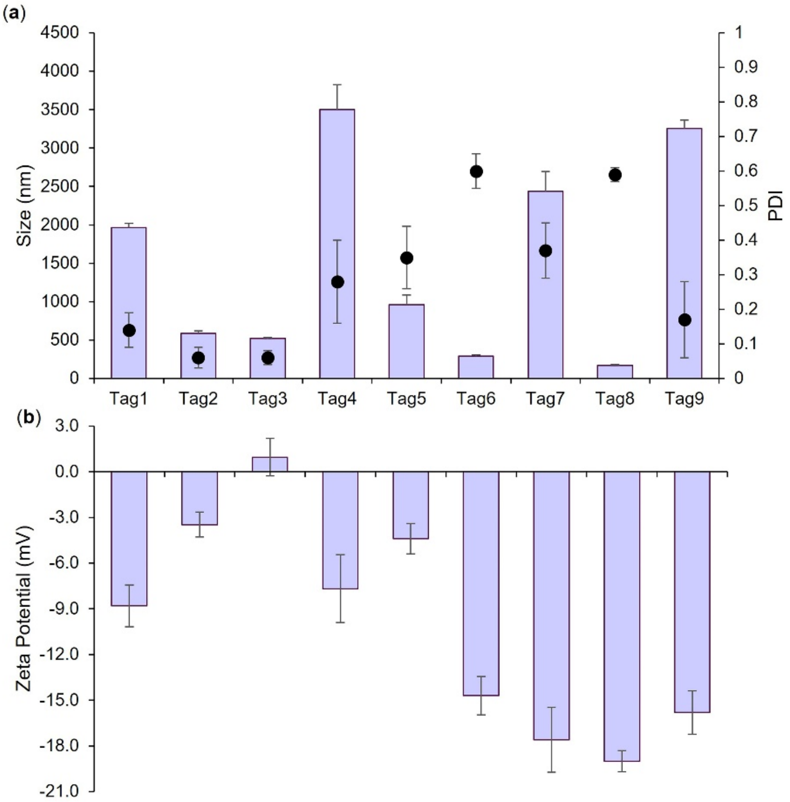

Figure S4. Size and PDI (**a**) as well as zeta potential (**b**) of 9 empty LPHNP formulations prepared according to a Taguchi L9 orthogonal array design of experiments (Table S2). Results represent mean ± SD of 3 technical replicates, as an initial screening study.

The initial screening study showed a collective tendency for formulations manufactured using the lowest employed lipid concentration of 2 mg/mL (Tag1-Tag3) to have a smaller size and PDI than those produced using 4 and 8 mg/mL of lipid (Figure S4). In light of this finding, a further screening study was conducted to investigate the impact of lowering the lipid concentration further, while also employing a range of FRR and two different TFR levels (Figure S5).

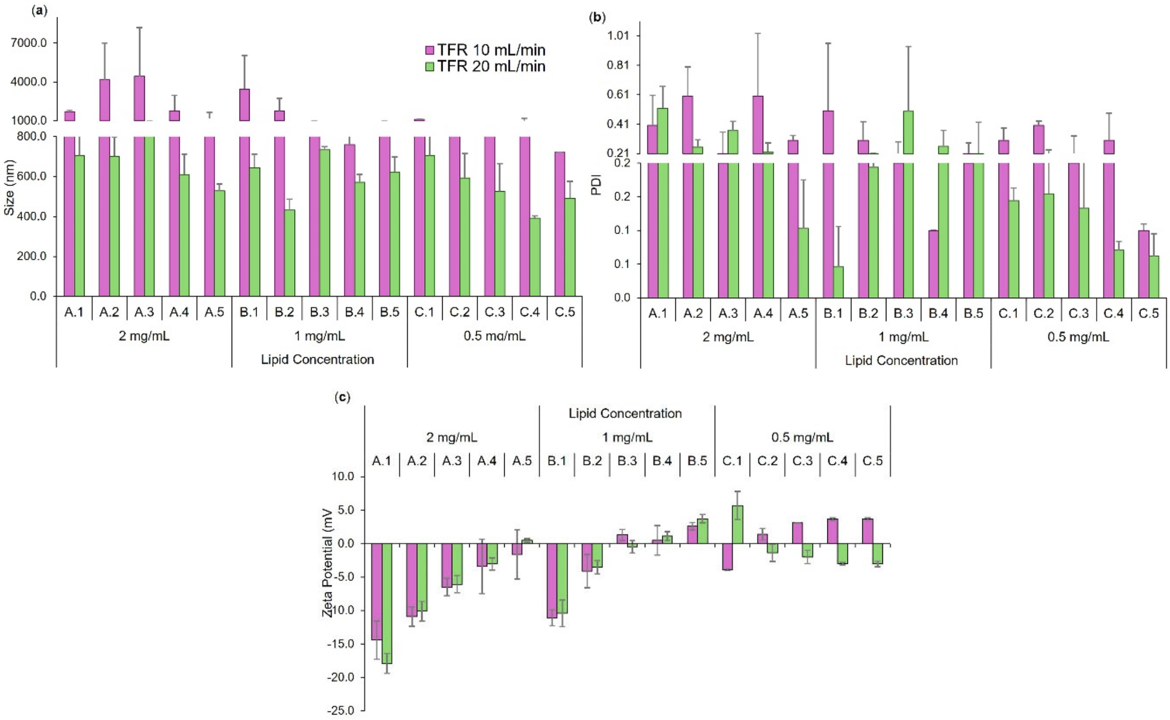

Figure S5. Size (**a**), PDI (**b**) and zeta potential (**c**) of 30 empty LPHNP formulations prepared using the full factorial design detailed in Table S3. Results represent mean ± SD of 3 technical replicates, as a second screening study.

A trend was observed for smaller and more uniformly sized empty LPHNPs to be produced at the lowest employed lipid concentration of 0.5 mg/mL, and at a TFR of 20 mL/min ('C' series, Figure S5). This series of formulations was therefore investigated further in order to determine the optimal FRR parameter (see main manuscript, where individual formulations have been renamed as 'E' (empty LPHNPs) together with the employed FRR, i.e. E2:1-E6:1).

2.4 Statistical Analysis of Co-Loaded LPHNP Physicochemical Characteristics (Corresponding to Figures 3 and 4, Main Manuscript)

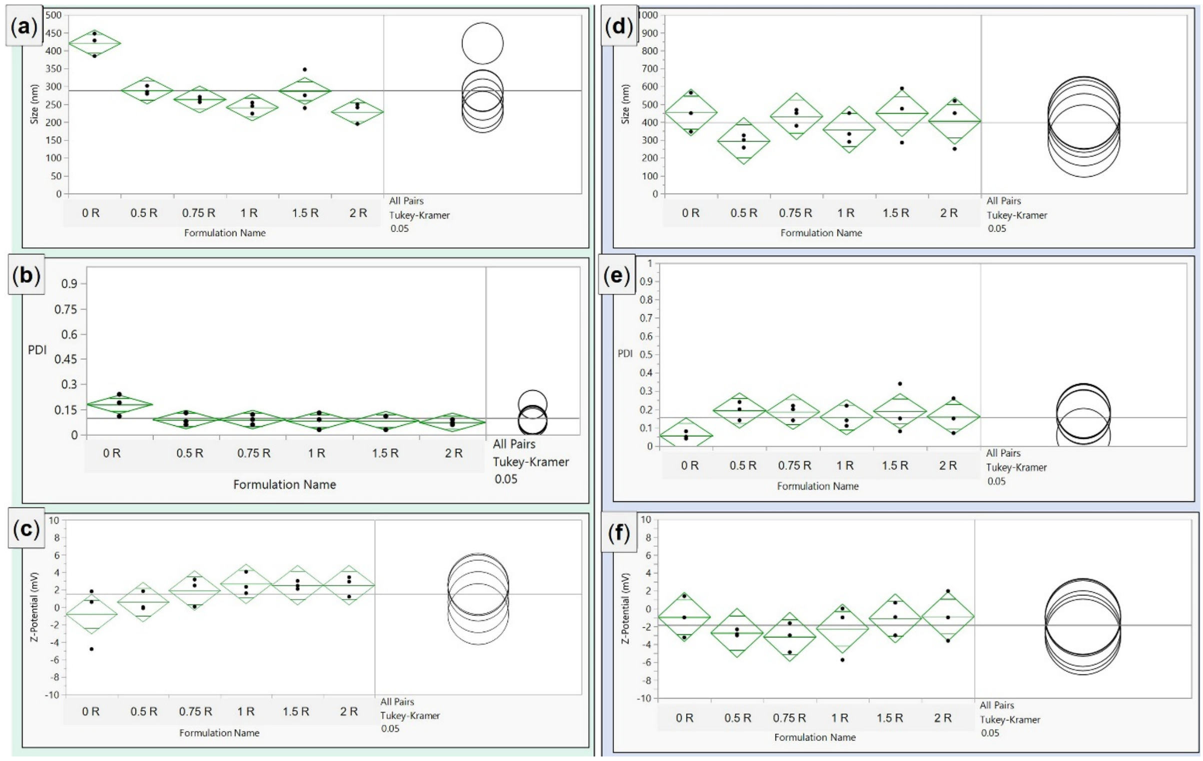

Figure S6. One-way ANOVA analysis for size (**a** and **d**), PDI (**b** and **e**) and zeta potential (**c** and **f**) with Tukey's post-hoc comparison performed on CTX and RN7IN6 co-loaded LPHNPs and comparator formulations measured immediately after production (**a**, **b** and **c**) and after purification by centrifugal ultrafiltration (**d**, **e** and **f**). Images are of analysis generated using JMP® 16.2.0 software. The horizonal line spanning the plot represents the mean of all datapoints combined. Green diamonds encompass datapoints for a formulation group (individually indicated by black dots), with the center line across each diamond corresponding to the group mean and the vertical span of each diamond representing the 95% confidence interval for each group. Diamond width and height is proportional to the group sample size, with broader diamonds indicating smaller sample sizes or higher variability. Black circles represent the mean values for each individual formulation – overlapping circles indicate a lack of significant difference between represented formulation groups, while circles that do not overlap indicate statistically significant differences in group means.

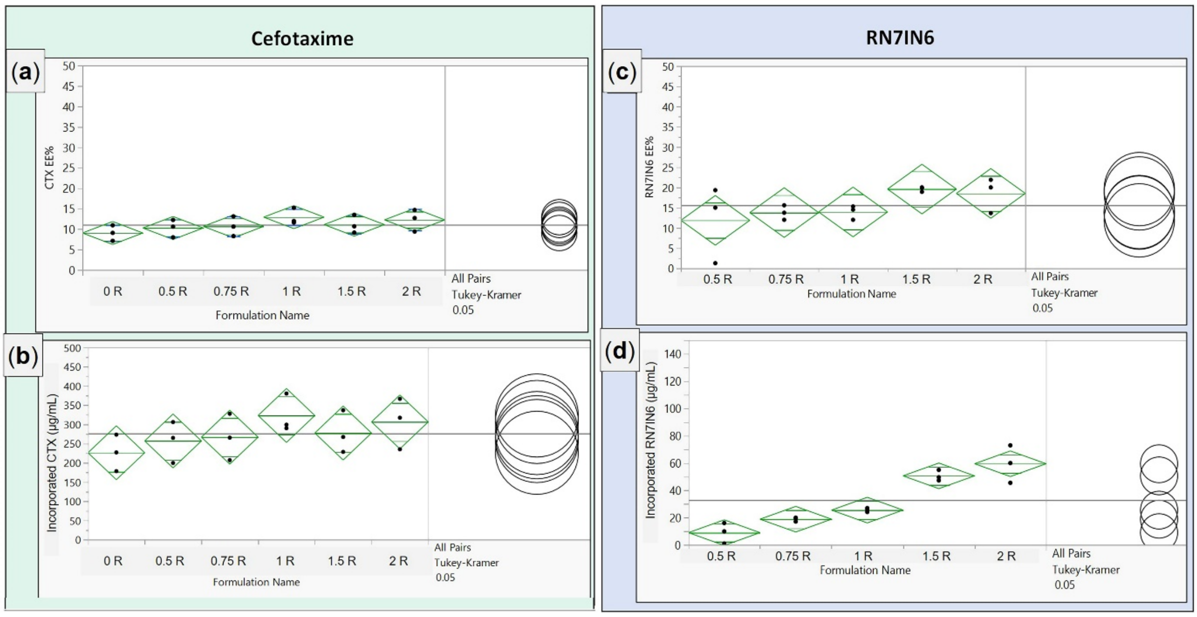

Figure S7. One-way ANOVA analysis for encapsulation efficiency (EE%) (**a** and **c**) and incorporated active concentration (**b** and **d**) with Tukey's post-hoc comparison performed on CTX and RN7IN6 co-loaded LPHNPs and comparator formulations for CTX (**a** and **b**) and RN7IN6 (**c** and **d**). Images are of analysis generated using JMP® 16.2.0 software. The horizonal line spanning the plot represents the mean of all datapoints combined. Green diamonds encompass datapoints for a formulation group (individually indicated by black dots), with the center line across each diamond corresponding to the group mean and the vertical span of each diamond representing the 95% confidence interval for each group. Diamond width and height is proportional to the group sample size, with broader diamonds indicating smaller sample sizes or higher variability. Black circles represent the mean values for each individual formulation – overlapping circles indicate a lack of significant difference between represented formulation groups, while circles that do not overlap indicate statistically significant differences in group means.
